# Reconstituted ferredoxin–MEP pathway of Apicomplexa in *Escherichiacoli* as an *in situ* screening platform for inhibitors and essential enzyme mutations

**DOI:** 10.1016/j.jbc.2025.110726

**Published:** 2025-09-16

**Authors:** Ojo-Ajogu Akuh, Deborah Maus, Martin Blume, Kevin J. Saliba, Frank Seeber

**Affiliations:** 1 FG16: Mycotic and Parasitic Agents and Mycobacteria, Robert Koch Institute, Berlin, Germany; 2 Department of Molecular Parasitology, Institute of Biology, Humboldt University Berlin, Berlin, Germany; 3 Research School of Biology, Australian National University, Canberra, Australia; 4 P6: Metabolism of Microbial Pathogens, Robert Koch-Institute, Berlin, Germany

**Keywords:** *Toxoplasma gondii*, redox regulation, *Plasmodium*, parasite metabolism, isoprenoid, iron-sulfur protein, flavodoxin, ferredoxin, apicoplast, MEP pathway

## Abstract

The apicoplast, an essential plastid-like organelle of apicomplexan parasites, including *Plasmodium spp*. and *Toxoplasma gondii*, harbors unique metabolic pathways absent in the host. Within the apicoplast, the ferredoxin redox system consists of plant-type ferredoxin-NADP^+^ reductase (ptFNR) and its redox partner, plant-type ferredoxin (ptFd). It donates electrons to the last two enzymes in the essential methylerythritol phosphate (MEP) pathway of isoprenoid biosynthesis. To establish an easy-to-handle platform for screening for enzyme inhibitors or functional mutations of the *Plasmodium falciparum* MEP pathway *in situ,* we established an *Escherichia coli* model where bacterial growth depended on the last enzyme IspH and its redox system ptFd and ptFNR. For this, we supplemented a flavodoxin and *ispH E. coli* double mutant with expression constructs for ptFd, ptFNR, and IspH from *P. falciparum*. These proteins could functionally replace the two essential endogenous *E. coli* enzymes, reconstituting the last step in the isoprenoid biosynthesis pathway of the apicoplast. To validate this strain as a screening platform, we used point mutations in ptFd as a surrogate for chemical pathway inhibitors. Several single mutants were evaluated by growth assays to identify amino acids that are essential for proliferation. We verified the mutants' consequences on the depletion of MEP metabolites by LC-MS analysis. Finally, some mutants were used to complement a conditional *T. gondii* Fd KO strain. The results mirrored those of the respective *E. coli* mutant, highlighting the model's utility in identifying functional mutations or ptFd/MEP pathway inhibitors before conducting more labor-intensive and time-consuming assays in parasites.

The phylum Apicomplexa comprises a diverse group of unicellular eukaryotic parasites of medical and veterinary importance, including *Plasmodium spp*. and *Toxoplasma gondii*, the causative agents of malaria and toxoplasmosis, respectively. A defining feature of these parasites is the presence of an apicoplast, a plastid-like organelle acquired *via* secondary endosymbiosis from red algae (1). This organelle plays a crucial role in essential metabolic pathways such as type II fatty acid biosynthesis and the methylerythritol phosphate (MEP) pathway of isoprenoid biosynthesis (2, 3, 4). The activity of some enzymes in these pathways depends on electrons supplied by a redox system consisting of the iron-sulfur cluster (ISC)-containing plant-type ferredoxin (ptFd) and its redox partner, a ferredoxin NADPH-dependent reductase (ptFNR) (5, 6). Electron flow occurs from NADPH *via* ptFNR to ptFd, which then transfers them one-by-one to acceptor proteins through protein–protein interactions (PPIs). Two recipient proteins of this electron transfer are 1-hydroxy-2-methyl-2-(E)-butenyl 4-diphosphate synthase (IspG) and 1-hydroxy-2-methyl-2-(E)-butenyl 4-diphosphate reductase (IspH), the two terminal enzymes of the essential MEP pathway (Fig. 1). This pathway produces the two isomeric end products isopentenyl pyrophosphate (IPP) and dimethylallyl pyrophosphate (DMAPP), the basic building blocks for other essential isoprenoids (7, 8). In *Plasmodium falciparum*, the first step of the MEP pathway can be inhibited by the drug fosmidomycin, leading to parasite death (9). Genetic studies in Apicomplexa harboring an apicoplast have shown that other enzymes of the MEP pathway are also required for proliferation (10, 11). *In vitro* experiments suggested that the pathway's last enzyme, IspH, receives electrons from *P. falciparum* ferredoxin (PfFd) (12). More recently, ptFd was shown to be essential for the survival of both *T. gondii* and *P. falciparum* (13, 14). Therefore, the MEP pathway and the associated ptFd/ptFNR redox system are considered prime drug targets, given their essentiality and absence of the enzymes in the host (5, 6, 15, 16).

**Figure 1:**
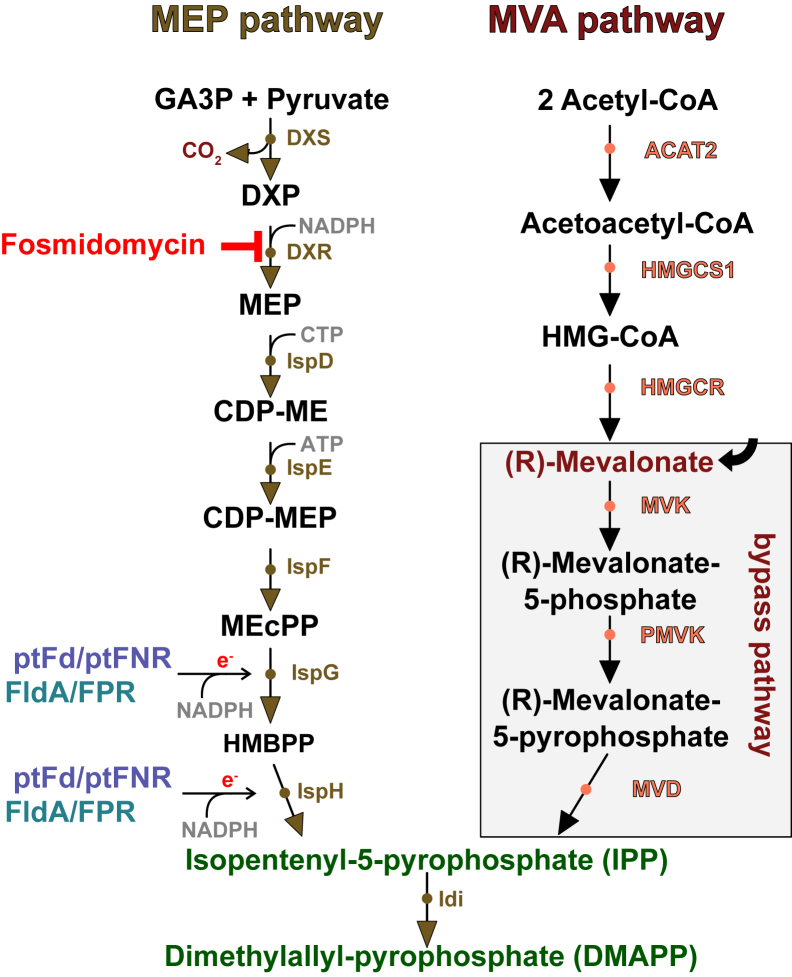
**Comparison of MEP and mevalonate pathway of isoprenoid biosynthesis.** The MEP pathway begins with glyceraldehyde-3-phosphate (GA3P) and pyruvate, while the mevalonate pathway (present in mammals) begins with two acetyl-CoA molecules. MEP pathway is present in both bacteria and the apicoplast and the redox systems capable of transferring electrons to IspG and IspH are shown (FldA/Fpr for bacteria and ptFd/ptFNR for the apicoplast). Fosmidomycin inhibits DXR. The plasmid-based, MVA-derived by-pass pathway consists of the three indicated enzymes plus Idi from *E. coli* as an operon. In the presence of mevalonate, IPP/DMAPP can be produced and ensures survival even in the absence of the MEP pathway. ACAT2, acetoacetyl-CoA thiolase; DXS, DXP synthase; DXR, DXP-reductoisomerase; IspD, 2C-methyl-D-erythritol 4-phosphate cytidyltransferase; DXP, 1-deoxy-D-xylulose 5-phosphate; FPR, ferredoxin/flavodoxin reductase; IspE, 4-diphosphocytidyl-2C-methyl-D-erythritol kinase; IspF, 2C-methyl-D- erythritol-2,4-cyclodiphosphate synthase; IspG, hydroxylmethylbutenyl diphosphate (HMBPP) synthase; IspH, HMBPP reductase; Idi, IPP isomerase; HMGCS1, HMG-CoA synthase; HMGCR, HMG-CoA reductase; MVK, mevalonate kinase; PMVK, phosphomevalonate kinase; MVD, diphosphomevalonate decarboxylase; MEP, methylerythritol phosphate; ptFd, plant-type ferredoxin; ptFNR, plant-type ferredoxin-NADP^+^ reductase; IPP, isopentenyl pyrophosphate; DMAPP, dimethylallyl pyrophosphate; FldA, flavodoxin A.

The MEP pathway used by many bacteria, algae, and also Apicomplexa can be regarded as an example of convergent evolution. Mammals rely exclusively on the very different mevalonic acid (MVA) pathway to produce IPP and DMAPP (Fig. 1), whereas plants possess both pathways. The difference in both, precursor metabolites and enzymes involved, has provided an opportunity to delete components of the MEP pathway and supplement it with components of the MVA pathway as transgenes plus mevalonate (Mev). This bypass thereby provides a controllable system to turn off essential genes involved in the pathway (Fig. 1), enabling the study of the role of individual components (10, 17, 18).

Seminal work by Puan et *al.* showed that in *Escherichia coli* the MEP pathway is the only pathway strictly dependent on the redox protein flavodoxin A (FldA) (19). A *fldA* gene knockout in a strain with a dysfunctional MEP pathway could only be complemented by providing a MVA bypass plasmid plus Mev in the growth medium, implying that no other redox system was able to complement FldA's function.

While numerous plant genes for IspH and a few for IspG have been shown to complement EcΔi*spH* or EcΔ*ispG* strains (see Table S1), whether the homologs from *P. falciparum* can do so has not been investigated (20, 21). Since the functional conservation between bacterial flavodoxin and apicoplast ferredoxin redox systems with respect to IspG and IspH is largely unexplored, a nonfunctional electron transfer between *E. coli* FldA and apicomplexan IspG/H could be a reason for this. Moreover, a recent study reported widespread incompatibilities of the two bacterial ISC synthesis machineries (*isc* and *suf* operons) in order to express functional IspG of different phylogenetic origin by providing a 4Fe-4S cluster (4-ISC), judged by complementation of an EcΔ*ispG* strain (22). Several enzymes in the apicoplast, including IspG and IspH, are labile ISC-containing proteins with a 4-ISC, requiring oxygen-free conditions for *in vitro* work. This restricts studies to labs which have experience and special equipment to work under such conditions (23, 24). Therefore, we have previously advocated the use of *E. coli* as a surrogate system for the apicoplast, in particular for PPI studies of ISC proteins (25). Previous work also showed that apicomplexan 4-ISC–containing lipoic acid synthase (LipA) can complement an *E. coli* Δ*lipA* strain (26, 27), suggesting that, in principle, ISC loading onto apicomplexan enzymes is feasible in *E. coli*.

Reverse genetic tools in *T. gondii* and *P. falciparum* require lengthy procedures to obtain stable clones, which can take several weeks for *P. falciparum*. Given the strong interest in the apicomplexan MEP pathway as a drug target (15, 16), an easier-to-handle platform for screening functional mutations and drug effects to complement complex and time-consuming *in situ* studies in the parasite would be of great benefit to identify candidate inhibitors. In this study, we set out to establish an *E. coli* model that could be used as a surrogate of the apicoplast MEP pathway and its accessory redox system.

## Results

### Apicomplexan *ispH* complements *E. coli* Δ*ispH* strain

No complementation attempts of an EcΔ*ispH* strain with any apicomplexan *ispH* gene have previously been published. We wondered if this might be due to EcFldA being unable to interact with PfIspH, given the fact that apicomplexan IspH proteins are on a different branch in a phylogenetic tree compared to the plant enzymes, with proteobacteria positioned between them (Fig. S1*A*). Our phylogenetic analysis is consistent with recently published data (1), indicating that *ispH* gene transfer from an ancestor of the Planctomycetota, Verrucomicrobiota, Chlamydiota superphylum rather than cyanobacteria took place. While cyanobacterial FldA is known to interact productively with ptFNR, a Planctomycetota, Verrucomicrobiota, Chlamydiota ancestry of IspH might be a problematic factor for complementation competency, even more so since Chlamydiae seem not to possess an *fldA* homolog (28).

Proteins destined for the apicoplast require an N-terminal so-called bipartite targeting domain (BTD), consisting usually (but not always, for example PfIspH) of a classical signal peptide, cleaved off in the endoplasmic reticulum. It is followed by a transit peptide for targeting the protein from there to the apicoplast. There it is usually also cleaved off to yield the mature protein, although a deviation from this rule has recently been described (29). The transit peptides differ in length and composition not only between proteins but also between *P. falciparum* and *T. gondii*, making predictions of cleavage sites difficult. We therefore used EcIspH's start methionine as a reference point for an active enzyme and depleted its long N-terminal part (219 amino acids; Fig. S1*B*). This version of PfIspH had been shown previously to be enzymatically active (12), and it was also used for the experimental determination of PfIspH's 3D structure (21).

**Figure 2:**
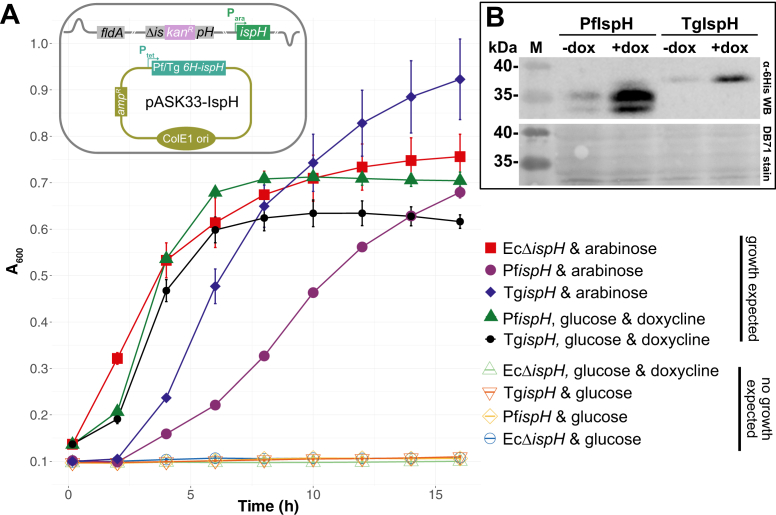
**Pf/Tg*ispH* complement *E. coli ispH* KO strain.***A*, growth curve of *E. coli* under different test conditions. Complementation is confirmed by growth in the presence of glucose/absence of arabinose and addition of doxycycline. Values are averaged from three independent experiments (n = 3), each carried out in triplicate. Error bars represent SD and are not visible if smaller than the symbols. The *cartoon* depicts the genetic makeup of the MGΔLy strain transformed with the doxycycline-inducible expression plasmids. *B*, Western blot analysis using an anti-6His-tag antibody shows expression of both Pf/Tg*ispH* under induced conditions (*top*). Note some leaky expression in the absence of doxycycline/presence of arabinose (dox). Total protein stain served as loading control (*bottom*).

To test for complementation by the *P. falciparum ispH* homolog, we used the previously described EcΔ*ispH* strain MGΔLy (30). It contains an arabinose-inducible, genome-integrated copy of Ec*ispH* on a kanamycin-disrupted Δ*ispH* background, making its growth arabinose-dependent (Fig. 2*A*, cartoon). Upon removal of arabinose and supplementation with glucose (for catabolite repression), MGΔLy can only grow when a functional IspH protein is provided *in trans*. Notably, expression of the N terminally 6×-His-tagged short PfIspH protein was readily able to complement MGΔLy when Ec*ispH* expression was suppressed and Pf*ispH* expression induced by adding doxycycline (Fig. 2*A*). A similarly shortened *T. gondii ispH* construct also supported growth of MGΔLy under these conditions. Protein production was corroborated by Western blot analysis, showing the presence of the proteins at the expected sizes (Fig. 2*B*). No growth was observed when MGΔLy transformed with the empty vector was maintained under the same condition. These results show that Pf/Tg IspH are functional enzymes even when a large portion of their respective N terminus is deleted and that they can complement an EcΔ*ispH* strain. It also means that EcFldA or another redox protein is a competent electron donor for apicomplexan IspH.

### *E. coli* flavodoxin is the electron donor required for the complementation of Ec*ΔispH* with apicomplexan *ispH*

**Figure 3:**
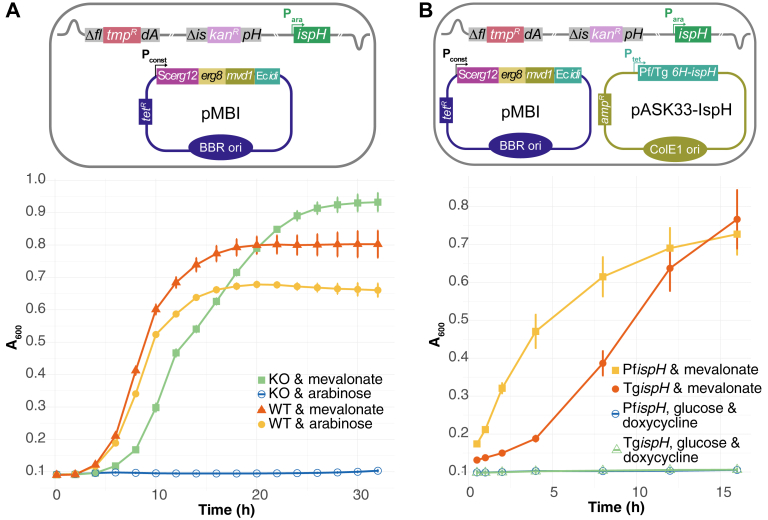
**Phenotypic confirmation of MGΔLy *fldA* knockout.***A,* growth curve of WT and double KO strains in the presence of mevalonate or arabinose. *B*, growth curve of EcMP2 under different test conditions. The involvement of Ec*fldA* is confirmed by the absence of growth despite the presence of glucose and doxycycline (presence of IspH). Values in both (*A*) and (*B*) are averaged from three independent experiments (n = 3), each carried out in triplicate. Error bars represent SD and are not visible if smaller than the symbols. The *cartoons* show a schematic representation of EcMP2 and the indicated plasmids used. FldA, flavodoxin A.

The data shown in Figure 2 are consistent with apicomplexan IspH proteins being “flexible” enough to interact with a redox enzyme of *E. coli* which has to provide two electrons for catalysis. The most likely candidate is EcFldA (19). To determine whether this is indeed the case, we decided to knock out Ec*fldA* in MGΔLy. However, since the protein is essential for isoprenoid biosynthesis and survival of *E. coli*, we first introduced a Mev by-pass pathway on a plasmid (pMBI; (31)) into MGΔLy. We then generated a knockout of Ec*fldA* using homologous recombination (32, 33) by insertion of a trimethoprim-interrupted *fldA* gene from strain KM20 (19) (Fig. 3*A*, cartoon). Correct replacement was confirmed by analytical PCR/sequencing of the targeted locus (Fig. S2). The generated strain was named EcMP2 and its growth phenotype evaluated (Fig. 3*A*). Unlike the wt, EcMP2 could not grow in the presence of arabinose, supporting the involvement of FldA in transferring electrons to IspH, as expected (Fig. 3*A*).

To further confirm the involvement of EcFldA, we transformed EcMP2 with expression plasmids for TgIspH and PfIspH, respectively (Fig. 3*B*, cartoon). In contrast to the experiment shown in Figure 2*A* where EcFldA was present, no growth was observed when Pf/Tg*ispH* were induced with doxycycline and EcIspH depleted (by absence of arabinose/presence of glucose) (Fig. 3*B*). Altogether, these results indicate the involvement of EcFldA as the electron donor for the apicomplexan IspH.

### Apicoplast Fd/FNR redox system and PfIspH can rescue EcMP2 growth

**Figure 4:**
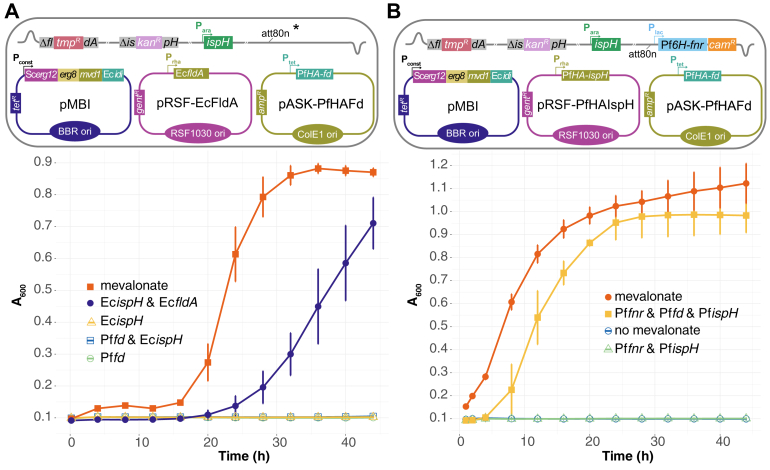
**PfFd, PfFNR, and PfIspH can functionally replace EcFldA and EcIspH.***A,* growth curves of EcMP2 showing that PfFNR is required for a functional apicoplast redox system in EcMP2. Note that in this experiment the strain does not contain the genome-integrated Pf*fnr* gene (∗, *cartoon*). Results from two independent experiments (n = 2) are shown, each conducted in triplicate. Error bars represent SD. *B*, growth curves of EcMP2-FH transformed with plasmids expressing Pf*ispH* and Pf*fd* (wt). Complementation is only seen when Pf*fnr*, Pf*fd,* and Pf*ispH* are expressed together. Growth in the presence of mevalonate served as positive control. Values are averaged from three independent experiments (n = 3), error bars represent SD and are not visible if smaller than the symbols. The *cartoons* show a schematic representation of EcMP2 and the indicated plasmids used. PfFd, *Plasmodium falciparum* ferredoxin; Pf*fnr, Plasmodium falciparum* ferredoxin-NADP^+^ reductase.

Having established an EcΔ*ispHΔfldA* strain, we tested whether PfFd and *P. falciparum* ferredoxin-NADP^+^ reductase (PfFNR) can substitute EcFldA. When PfFd was present alone, EcMP2 was unable to grow but required the expression of Ec*fldA* from another plasmid (Fig. 4*A*, cartoon and growth curve), indicating that none of the endogenous *E. coli* reductases was able to provide electrons to PfFd. However, introducing Pf*fnr* into the genome *via* the *att80n* phage attachment site led to the growth of EcMP2-Pf*ispH*-Pf*fd* (Fig. 4*B*). Again, the entire ptFd/FNR redox system was required since Pf*fnr* alone was insufficient for growth (Fig. S3). Taken together, these experiments confirmed that PfFNR's specific interaction with PfFd is nonredundant and that both are required for complementation in the absence of EcFldA. EcMP2 containing Pf*fnr*-Pf*ispH* was called EcMP2-FH, and EcMP2-FHF when it also contained Pf*fd*_wt_ or mutant (*e.g.,* EcMP2-FHF_C44A_).

### Some mutations in the interaction interface between PfFd and PfIspH lead to growth arrest of EcMP2-FHF

Strain EcMP2-FHF can potentially be useful as a surrogate for the final part of the apicomplexan MEP pathway, for example, for testing inhibitors of any of the three proteins' functions. As a proof of concept, and in the absence of known specific and readily available chemical inhibitors (34), we wanted to test how this strain responds to changes in amino acids that were previously suggested to be important for the interaction between PfFd and PfFNR. Their mutation should lead to the interruption of electron flow, consequently to declined or halted growth of EcMP2-FHF and thus mimic drug-like effects. Of equal importance, EcMP2-FHF could be useful for further studies aimed at identifying amino acids involved in PPI. Fine-mapping of these regions could help in predicting binders to PPI interfaces (compounds or peptides).

**Figure 5:**
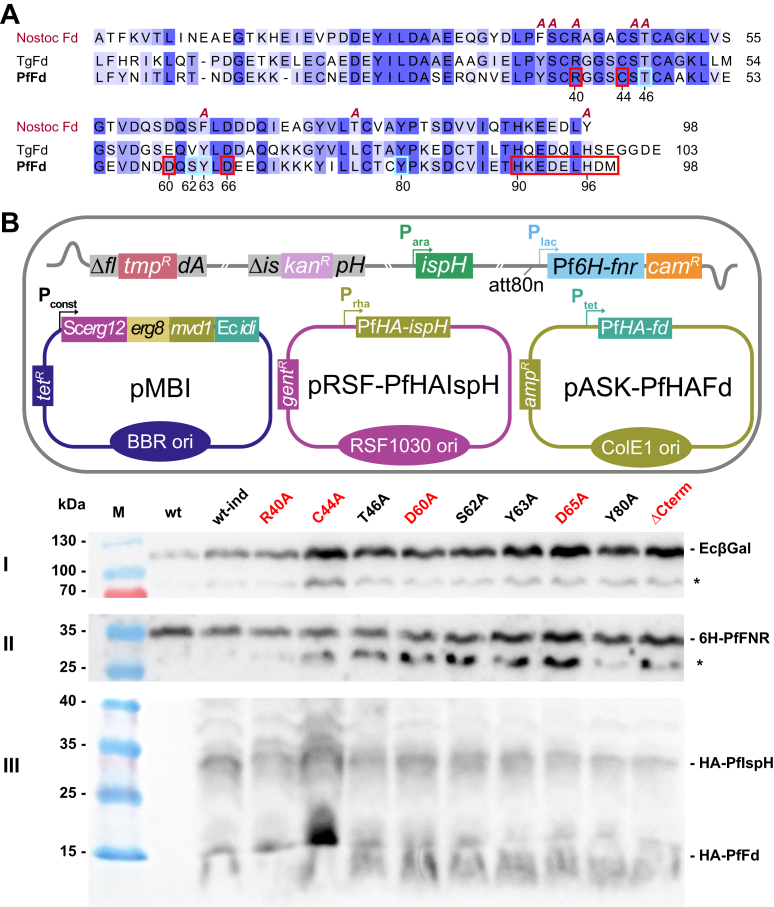
**Effect of select mutations at the predicted interaction interface between PfFd and PfIspH in EcMP2-FH***. A*, sequence alignment between PfFd and TgFd (without BTD) in comparison to cyanobacterial ptFd from *Nostoc* sp. (P0A3C7; previously named *Anabaena*; 55% identical to PfFd). The A's on *top* of the Nostoc sequence indicate the positions of published amino acid-to-Ala mutations (39). Numbered amino acids are mutated in PfFd (*red box*, shown to be essential for function; *light blue box*, not essential), with the exception of the C-terminal end (aa 90–98), which was deleted by introducing a stop codon at aa 90. *B*, Western blot of individual strains containing the indicated mutants after expression induction with IPTG, rhamnose, and doxycycline. Anti-6His antibody was used to detect PfFNR (II), whereas anti-HA antibody reacted with both, PfIspH and any of the PfFd mutants (III, and sketch on *top*). Note that Pf*fnr* expression from the lac promoter is leaky. Growth medium contained mevalonate, and β-galactosidase served as loading control (stained with a specific monoclonal antibody, I). ∗ presumably degradation products. The *cartoon* on *top* shows a schematic representation of EcMP2 and the indicated plasmids used. PfFd, *Plasmodium falciparum* ferredoxin; Pf*fnr, Plasmodium falciparum* ferredoxin-NADP^+^ reductase; BTD, bipartite targeting domain; ptFd, plant-type ferredoxin.

Based on our own analysis of PPI with AlphaFold 3 (AF3) (35) or Chai-1 (36) models (see Experimental procedures and Figures S4 and S5 for details and discussion) and previous studies (21, 37), some amino acid residues in PfFd were selected for site-directed mutagenesis (Fig. 5*A*). We opted for their replacement by alanine because it does not impose extreme steric or electrostatic effects on the structure (38). While this might not be true in all cases, extensive mutagenesis has been performed for cyanobacterial Fd (55% identical to PfFd), including Ala substitutions (39) (Fig. 5*A*). In most cases (with the exception of Cys mutants being part of the 2-ISC) Ala mutations had little effect on overall structure. Ala substitutions might thus complement previous studies with PfFd-interacting PfFNR where amino acids of opposite charge were mostly used (40).

**Figure 6:**
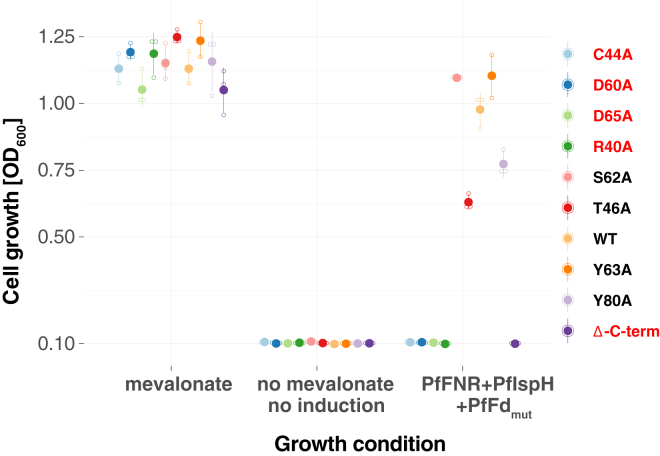
**EcMP2-FH can be used to screen for loss of function of PfFd.** Summary of the complementation competency of PfFd mutants. The plot is based on end-point measurements of individual growth assays at A_600_ nm under the indicated growth conditions. *Solid circles* show the mean from three independent experiments (*small circles*), error bars represent SD and are not visible if smaller than the symbols. Mutations resulting in no growth are indicated in *red*. Growth curves for each of the single mutants are shown in Figure S6. PfFd, *Plasmodium falciparum* ferredoxin.

EcMP2-FH was transformed with the respective PfFd mutant-expressing plasmid and tested for complementation by growth assay in liquid culture with wt PfFd (PfFd_wt_) as positive and PfFd_C44A_ as negative control. Cysteine 44 is part of the essential 2-ISC of PfFd (Fig. S5), and thus the mutation will result in a nonfunctional electron carrier and presumably also destabilize the protein to some extent (39). All resulting mutant strains were tested by Western blot with anti-tag antibodies for the expression levels of the three parasite proteins (Fig. 5*B*). Overall, the protein amounts of mutant PfFds were comparable, and growth phenotypes observed in parallel did not correlate with fluctuations in signal strength (Fig. 5*B*). When tested under inducing conditions, the growth assays showed that essential residues are R40, D60, D65, and C-terminal deletion (PfFd H90 residue replaced with a stop codon; Fig. 6). In contrast, T46A, S62A, Y63A, and Y80A mutants seemed not to influence electron flow to an extent that would inhibit growth (Figs. 6; S6). Taken together, EcMP2-FHF proved useful for the identification of PfFd point mutations resulting in either a severe or no growth defect. This strain can therefore be used as a screening platform for chemical inhibitors of the three apicomplexan proteins.

### Metabolic changes of the PfFd mutants reflect their impaired growth phenotype in *E. coli*

**Figure 7:**
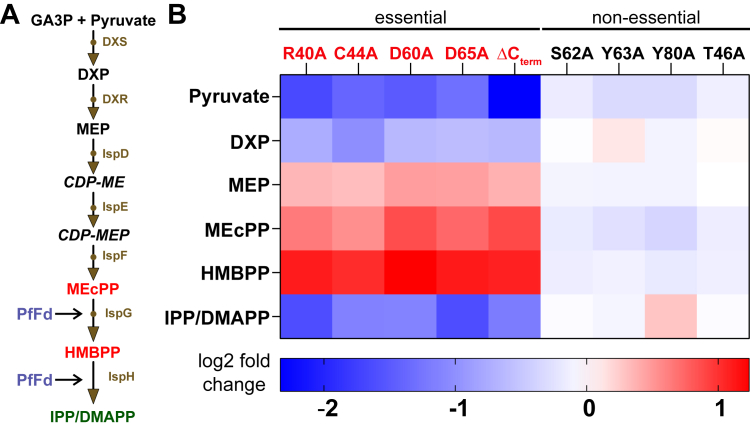
**Metabolomics analysis of PfFd mutants in *E. coli*.***A*, MEP pathway showing the steps directly affected by PfFd mutants highlighted in *red*. Metabolites not analyzed are 4-diphosphocytidyl-2-C-methylerythritol (CDP-ME) and CDP-ME phosphate (CDP-MEP). *B*, heat map of log2-fold change compared to PfFd_wt_, showing a change in the analyzed metabolites of individual “essential” PfFd mutants compared to “nonessential” PfFd mutants (analyzed by LC-MS). Shown is the result of one of two experiments with similar outcome, each consisting of four technical replicates. PfFd, *Plasmodium falciparum* ferredoxin; MEP, methylerythritol phosphate.

To test whether metabolite patterns in the MEP pathway would match the growth behaviour of the mutants, we sampled equal amounts of cells from each strain and analyzed the relative content of respective metabolites by a targeted LC-MS assay (see Experimental procedures for details). We observed a distinct metabolic profile based on the metabolites analyzed (Fig. 7*A*), differentiating the essential and nonessential PfFd mutants as reflected by the accumulation of MEcPP and HMBPP relative to the pathway's end product DMAPP/IPP in strains with mutated amino acids essential for growth (Fig. 7*B*). The nonessential mutants showed comparable metabolomic profiles to PfFd_wt_, as expected. These results are consistent with a block in the MEP pathway due to nonfunctional IspG and/or IspH (Fig. 7*A*).

### PfFd_mut_ phenotypes can be validated in the apicoplast of *T. gondii*

So far, none of the amino acids implicated in PfFd PPIs with PfIspH or PfFNR (21, 41) or predicted here have been verified in the parasite. We were therefore interested to know whether similar functional phenotypes would be observed when PfFd_mut_ is expressed in the apicoplast. Making transgenic parasites is more straightforward and rapid in *T. gondii* than in *P. falciparum*. From previous two-hybrid data, it was known that TgFNR and PfFd interact with each other (42), as well as PfFd with TgLipA (25). We were therefore confident that complementation of our previously generated inducible knockdown of TgFd (TgiΔFd) in *T. gondii* tachyzoites (13) with PfFd_wt_ and PfFd_mut_ would provide a suitable system to validate the effect of the mutations upon their expression in the apicoplast.

**Figure 8:**
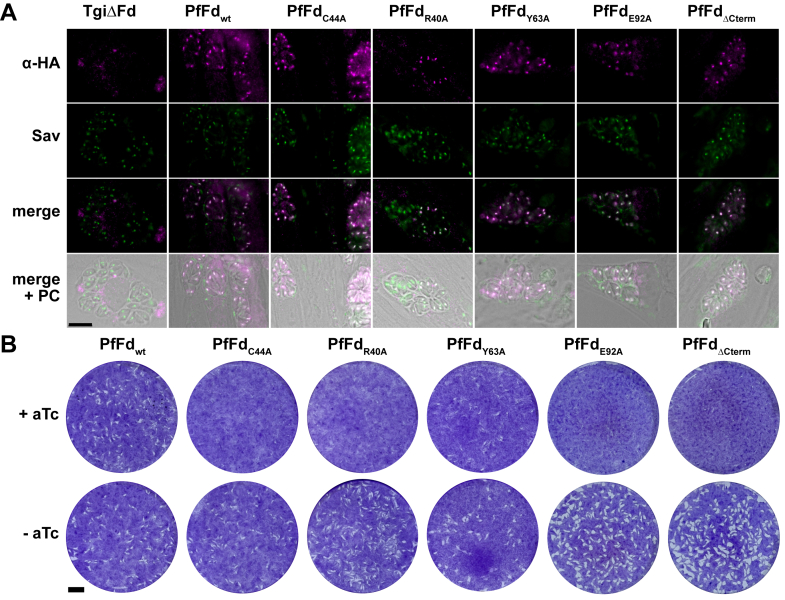
**Apicoplast localization and complementation of TgiΔFd with PfFd_wt_ and mutants.***A,* microscopy images showing localization of PfFd_wt_ and PfFd_mut_ to the apicoplast in respective knock-in clones (in the absence of aTc). Streptavidin (Sav) served as apicoplast marker, decorating biotinylated proteins there and (to a lesser extent) in the mitochondria of parasite and host (43). Overlapping fluorescence between HA-tagged PfFd and Sav results in a *white* signal in the merged images. Untransfected TgiΔFd served as negative control. Image brightness was adjusted to aid in the visualization of the apicoplast. The scale bar represents 10 μm. *B,* plaque assays measuring continuous proliferation of a TgiΔFd strain complemented with constructs of PfFd_wt_ or mutant, cultured in the absence (−) or presence (+) of anhydrotetracycline (aTc) for 8 days. Assays are from a single experiment out of three independent experiments, all with similar outcomes. The scale bar represents 5 mm. PfFd, *Plasmodium falciparum* ferredoxin; PC, phase contrast.

We focused on a subset of mutants and knocked-in PfFd-expressing plasmids to the nonessential UPRT locus of TgiΔFd using homologous recombination (Fig. S6). Immunofluorescence microscopy of the hemagglutinin (HA)-tagged proteins was used to confirm their expression and also their correct localization to the apicoplast by colocalization with apicoplast-resident biotinylated acetyl-CoA carboxylase (43) (Fig. 8*A*). We then followed the growth of the parasites over several lytic cycles by plaque assay after adding anhydrotetracycline (aTc) to the medium for 8 days, which causes TgFd's depletion (13). The presence of plaques indicated successful complementation with PfFd_wt_ (Fig. 8*B*), validating our assumption that PfFd works well in *T. gondii*. In contrast, the two essential mutants from the EcMP2 screen tested (R40A, ΔC-term) and E92A as a single mutant of a C-terminal residue (tested here but not in *E. coli*), as well as the PfFd_C44A_ mutant (predicted not to complement) did not show any plaques (Fig. 8*B*). With PfFd_Y63A_ we saw inconsistent results (small plaques under “plus aTc” condition) in three biological replicates, which can occasionally occur due to TgFd's relatively long half-life (13). However, it could also point to some complementation by this mutant, which has to be evaluated further. For technical reasons, *T. gondii* strains expressing PfFd_D65A_ and PfFd_T46A_ have so far not been obtained. Although they would have completed the picture in the parasite, their absence does not affect the overall conclusion that the growth phenotypes (*i.e.,* being able to complement or not) of the selected PfFd mutants seen in the *E. coli* model are similar to those of the apicoplast in *T. gondii.*

### Shared amino acids between *E. coli* and *P.falciparum* proteins involved in PfFd PPI

**Figure 9:**
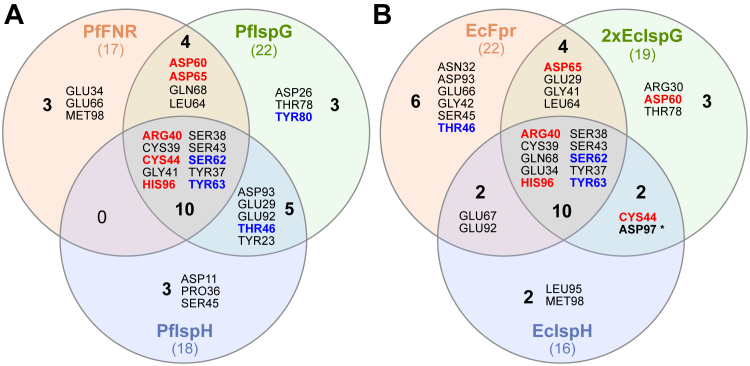
**Amino acids at interaction interfaces.***A,* Venn diagram showing PfFd residues at the interaction interface with PfFNR, PfIspG, and PfIspH. *B,* Venn diagram showing PfFd residues at the interaction interface with EcFpr, EcIspG, and EcIspH. In both (*A*) and (*B*), the nonessential residues in the *E. coli* model screen are highlighted in *blue,* while the essential ones are in *red*. PfFd, *Plasmodium falciparum* ferredoxin; Pf*fnr, Plasmodium falciparum* ferredoxin-NADP^+^ reductase.

Within the MEP pathway, Fd (or FldA) interacts not only with IspH but also with IspG and FNR. Consequently, it is not *per se* possible to assign an essential mutation to a singular specific PPI. Based on extensive structural modeling of all the constellations we confirmed previous suggestions that plant and *P. falciparum* IspGs (44) fold into a monomeric active structure containing a third domain (45) whereas EcIspG, like other bacterial IspG's, is a head-to-tail homodimer (see Figs. S8 and S9 for details and discussion). Three of the five essential PfFd amino acids identified (R40; C44; H96 as part of ΔC-term) are in close enough distance to interact with all three *P. falciparum* enzymes, whereas for D60 and D65, this is the case only for PfFNR and PfIspG but not for PfIspH (Fig. 9*A*). Likewise, all five amino acids are predicted to interact also with the *E. coli* enzymes, although with a slightly different distribution (Fig. 9*B*). Taken together, while these data provide further evidence that these amino acids are truly essential, they do not allow us to conclude which interaction(s) are responsible for failure to complement. This will require point mutations of the respective amino acids of the individual Fd-interacting protein. Due to the modularity of strain EcMP2-FHF, this can be readily accomplished.

## Discussion

Using *E. coli* or other engineered bacteria as a drug screening platform is not a new concept (46, 47) but it is gaining new interest due to methodological advances in high-throughput antimicrobial testing (48, 49). In this study, we engineered *E. coli* such that it becomes dependent on the function of three apicoplast proteins from the malaria parasite *P. falciparum*: ferredoxin, its reductase and IspH of the MEP pathway. These proteins are known to be essential and thus qualify as potential drug targets (5, 14). Moreover, a recent study identified PfFNR and members of the MEP pathway among the 27 high-priority candidates for antimalarial drugs in the whole *P. falciparum* proteome (50). Several studies have reported the use of *E. coli* for functional complementation of metabolic pathways it shares with *T. gondii* or *P. falciparum* (*e.g.* (2, 26, 27, 51, 52)). Advantages of an *in situ* assay are that compound entry into the cell, general cytotoxicity (in case of EcMP2-FHF in the presence of Mev) and specificity for the three enzymes (in absence of Mev but induction of transgene expression) are tested simultaneously (46). It can therefore serve as an initial means of qualifying or disqualifying a drug-like compound before further testing in the parasite.

One major reason for the development of the EcMP2-FHF strain was also the fact that PfFd and in particular PfIspH possess oxygen-labile 4-ISCs that are very difficult to work with *in vitro* unless special and expensive lab equipment is used (23, 53, 54). We focused on PfFd as the mutated protein due to its central role in the organelle's metabolism (5). The initial choice for PfIspH as an interacting partner for PfFd was based on our prior data showing that PfFd could act as electron donor for PfIspH *in vitro* (12) and the availability of an EcΔ*ispH* strain (30). It would be expected that the other Fd-interacting proteins should be suitable partners as well.

In our study, the readout to assess the importance of a PfFd mutation on *E. coli's* fitness was bacterial growth. However, we initially had difficulties in growing EcMP2-FHF upon removal of Mev and in the presence of all antibiotics. This was partly overcome by adding only the antibiotics required for plasmid maintenance (Tet, Gent, Amp), leaving out kanamycin, trimethoprim, and chloramphenicol. These three resistance genes are genome-integrated (Fig. 6*A*), thus their temporary omission during growth experiments is regarded as less problematic. While we have not analyzed the reasons for this effect further, it is interesting to note that mutations in *ispH* (previously also called *lytB*) lead to the activation of the so-called stringent response in *E. coli*, resulting in the synthesis of the alarmone guanosine tetra- or penta-phosphate ((p)ppGpp) by the enzyme RelA (55, 56). Under IspH-depleted conditions it can also occur in the absence of starvation, with multiple effects on metabolism and growth (57). There exist complex interactions between certain antibiotic ribosome inhibitors, including chloramphenicol, tetracycline, and trimethoprim and the relA-mediated stringent response, which could affect growth and reactive oxygen levels (58, 59). It might be worthwhile to redesign an EcMP2-FHF–like strain in this respect, for instance by generation of *fldA* and *ispH* deletions and Pf*fnr* insertion by CRISPR-Cas9–based methods that do not leave selection markers behind (60). However, as previously reported, the expression level of the MVA bypass seems to be crucial and requires a high copy number of the operon (61).

We showed that metabolomics can also be used to corroborate an observed phenotype in the MEP pathway by its metabolic footprint. Given the ease with which large amounts of bacterial cultures can be grown for this purpose, this is a further benefit of the model, in particular when assessing the specificity of effects upon exposure to potential inhibitors. Although the growth phase has substantial influence on *E. coli*'s metabolome, in particular under stressed conditions (62, 63), our data show that these requirements can be readily met in *E. coli* and with much less effort compared to *T. gondii* or *P. falciparum*, where low abundance of the MEP pathway metabolites require large quantities of cells. The observed metabolite abundance changes in the essential mutants are those expected from an *ispH* knockdown (Fig. 8) and are consistent with our previously reported results in the TgiΔFd strain (13) and also seen for a mutated *ispG E. coli* strain (64). Recently, it was shown that an increase in IPP/DMAPP levels in *E. coli* leads to monomerization of DXP synthase, the first enzyme in the MEP pathway, thereby decreasing its activity (65). Whether such a negative feedback mechanism also works in case of HMBPP accumulation (*e.g.* caused by inhibition of IspH activity) needs further investigation. It would explain the lower DXP levels seen in the essential PfFd mutant strains (Fig. 7).

In our proof-of-concept study, we used mutant PfFd more as a surrogate for chemical inhibitors of PfIspH to validate EcMP2-FHF as a screening platform. However, this strain is also very useful for PPI studies, in particular when combined with AF3/Chai-1–based models and their verification by simple mutagenesis experiments. Some findings in this context are addressed below (see Figs. S5 and S8 for further discussion).

Based on prior information, successful functional replacement of EcIspH and EcFldA by PfIspH and PfFd/FNR was in several ways surprising. A previous report implied that *ispG* from *P. falciparum*, but also from diverse bacterial species, was unable to complement a Δ*ispG E. coli* strain (see Supplementary Materials in (22)). The authors attributed it to incompatibilities of the bacterial ISC synthesis machinery and its associated electron carrier proteins to properly load ISCs onto the investigated IspGs of different phylogenetic origin. It implied that this could be a general problem and also affect PfIspH. However, our demonstration that Pf/TgIspH is able to complement MGΔLy indicates that ISC loading onto these two proteins functions properly. Previous studies showed that *E. coli* depleted of the entire *suf* system (involved in ISC biosynthesis besides the *isc* gene cluster) still grew like wt and without a requirement for a MVA bypass (61, 66). Moreover, SUF proteins were only found in the proteome of single colonies cultured on solid media but undetectable in liquid cultures of *E. coli* (67). This indicates that *E. coli* IspG and IspH, the only two indispensable ISC-containing proteins, can obtain their 4-ISCs also *via* the ISC proteins of the *isc* gene cluster under the right conditions. This reflects the redundancy but also flexibility of both ISC systems in terms of loading of clusters to “foreign” proteins. At least for Pf/TgIspH this is the case, as shown here (Fig. 2).

We would also expect that electron transfer to PfIspG works in a Δ*ispG* strain similar in setup to EcMP2. We noticed, however, that the protein sequence for recombinant PfIspG expression used by D'Angelo *et al.* still contained the N-terminal BTD, as well as additional amino acids, which we considered unnecessary in a bacterial context (22) (Fig. S8*B* for IspG), and similar to PfIspH (see Introduction). *P. falciparum* and *T. gondii* are known to contain substantial numbers of large insertions within proteins (68), with mostly unknown functions or consequences. In the case of PfIspG, the extended N terminus might negatively influence PPIs with PfFd, leading to PfIspG's inactivity (Fig. S8*C*). A similar analysis for PfIspH is shown and discussed in Figure S9. This assumption is consistent with the successful complementation of an EcΔ*ispG* strain by two plant IspG proteins, depleted of their N-terminal chloroplast targeting signals (69, 70), and which also have a similar predicted AA∗B monomeric structure like PfIspG (Fig. S8*A*; (44)).

Another factor of uncertainty in the context of N-terminal extensions was that complementation of EcΔ*ispH* by *ispH* from various plants (there called HDR) and cyanobacteria required further N-terminal sequences (called N-terminal conserved domain, NCD) beyond the start methionine of EcIspH (which we used as reference point for our construct) (71). It has been suggested that the NCD in those species may be involved in protecting them from high oxygen concentrations during photosynthesis (72). However, the free-living relatives of Apicomplexa, the photosynthetic Chrompodellids, do not show a similarly conserved NCD (Fig. S1*B*). Moreover, despite being >85% identical to their respective complementing IspH isoform from the same species, two plant proteins containing the NCD did not complement an EcΔ*ispH* strain (73). Along those lines, conserved hydrophobic amino acids in the C-terminal part of IspH of many species (Y198 and F302 in *E. coli*; F replaced by W in Apicomplexa) have been shown to prevent EcIspH from oxidative damage (44). However, the cyanobacterial and plant IspH's have much shorter C termini, lacking F/W302 (Fig. S1*B*). Taken together, predictions of functional consequences on IspH based on simple sequence comparisons should be considered cautiously. Our EcMP2 strain might serve as a platform to test the general importance of N- and C-terminal extensions and suspected “key residues” in IspH and IspG from Apicomplexa but possibly also from plants or cyanobacteria.

One of the notable findings in this study is that despite the structural differences between ptFd and EcFldA, they are still able to complement each other in *E. coli.* This might have been expected due to several variable segments along the sequence of ptFds that provide a level of structural flexibility allowing different small regions to engage in different PPIs (74, 75). It will be interesting to see whether this flexibility also extends to flavodoxins from bacteria and if they can replace Fd in Apicomplexa as well. From prior extensive knowledge of PPIs between ptFd and the diverse array of interacting proteins, including those of photosystem I (76, 77), it is unsurprising that electrostatic interactions also appear to play a major role whether a particular PfFd mutant is essential or not. It is in line with previous *in vitro* data showing that amino acids of opposite charge are involved in the contact and electron transfer between PfFd and PfFNR (37, 41, 78). In general, such interactions are a prerequisite for transient, weak protein–electron donor interactions and also partly explain the promiscuity of ptFd and FldA in their choice of electron acceptors. However, it seems not to include any of the bacterial NADP-dependent reductases since PfFNR was required for complementation. Whether this dependence lies in some catalytic peculiarities of ptFNRs compared to EcFpr, as described recently (79), needs to be determined.

Most available experimental data are for interacting residues between PfFd and PfFNR, the least challenging proteins with respect to ISC's sensitivity to oxygen. They have relied on first expressing and purifying a mutant protein in *E. coli* and then investigating the protein's effects on electron transport and function *in vitro* in order to predict a resulting fitness effect of the mutation (34, 80, 81, 82, 83). The *in vitro* experiments have the advantage of providing detailed biochemical and mechanistic data, whereas our approach tests a mutant's phenotype directly *in situ.* It is thus complementary to those studies and is facilitated by the fact that both expression plasmids in EcMP2-FHF can directly be used for large scale protein expression and purification upon adaptation of the detection tags used here. The final judgement of a mutation can then be done in an inducible knockdown strain similar to TgiΔFd, as shown here, or by complementation of a PfFd KO strain (14).

In conclusion, while in the past PPI have been regarded as poorly druggable by drug-like substances, this view is shifting with the powerful tools of AI to predict binders to PPI interfaces that could thus disrupt the protein's functions (84, 85). The ease with which such PPI “interrupters” can be introduced into and screened within a strain like EcMP2-FHF might thus lead to a novel class of inhibitors, beyond those already present in existing large compound libraries. The task is to find them.

## Experimental procedures

### Bacterial and parasite strains, plasmids, chemicals

For general cloning purposes, *E. coli* NEB 5*α* competent cells (New England Biolabs; NEB) were used. The EcΔ*ispH* strain MGΔLy was described previously (30) and obtained from the *E. coli* Genetic Resource Center (https://ecgrc.net). Strain KM20 was provided by Craig T. Morita. The cloning vector pICOz (86) was purchased from BCCM/GeneCorner Plasmid Collection (https://www.genecorner.ugent.be). pMBI (carrying the Mev by-pass operon (31), was a gift from Jay Keasling (Addgene plasmid #17816), and pSAG1::CAS9-U6::sgUPRT a gift from David Sibley (Addgene plasmid #54467). pKD78 (32) was obtained from the *E. coli* Genetic Resource Center. Immortalized human fibroblast cell line BJ-5ta (CRL4001) was obtained from American Type Culture Collection. Cultures were regularly tested for absence of microbial contamination using PlasmoTest (InvivoGen).

Generation of *T. gondii* RH strain TgiΔFd has been reported previously (13). Restriction enzymes, competent cells, and NEBuilder HiFi DNA Assembly kit for Gibson cloning were from NEB. Chemicals and bacterial growth media components were from Carl Roth, Merck/Sigma or Thermo Fisher Scientific, unless otherwise stated.

### PCR and sequencing

Genomic DNA (gDNA) was extracted from parasites for screening purposes by using DNAzol reagent (Invitrogen). All PCRs were conducted by using either Q5 Hot Start High-Fidelity DNA polymerase (New England Biolabs) for cloning purposes, or DreamTaq (Thermo Fisher Scientific) for diagnostic/screening purposes. Correctness of DNA constructs, plasmids, and PCR products from genes/regions of interest in mutant parasites were verified by Sanger sequencing using the primers listed in Table S2.

### Genetic constructs

Unless stated otherwise, DNA cloning was performed using HiFi DNA Assembly kit. For complementation of EcΔ*ispH*, the following doxycycline-inducible expression constructs were made. To make 6x-His tagged Pf/TgIspH, TgIspH (ID TGME49_227420) was amplified with primers 1 and 2 (Table S2) using gDNA of *T. gondii* tachyzoites (RH strain) as template (excluding the BTD sequence; Fig. S1), and combined with the pASK33plus vector (IBA GmbH, amplified with primers 3 and 4). HA-tagged PfFd was amplified (from an existing pASK33plus-6His-PfFd plasmid) with primers 5 and 6 and combined with pASK33plus. The resulting plasmid was used as a template to generate PfFd mutants by site-directed mutagenesis using the primers 8 to 25 and Q5 site-directed mutagenesis kit. All the constructs were verified by Sanger sequencing with primer 7.

For the rhamnose-inducible Pf*ispH* construct, the arabinose promoter of pBAD1030G (87) was replaced with the rhamnose promoter from pAvi-ccdB (88) by digesting pBAD1030G with NsiI and NheI and combined with the PCR-amplified pRHA-ccdB fragment (primers 26 and 27), resulting in pRHA1030G-ccdB. The *ccdB* fragment was then replaced with PCR-amplified, codon-adapted Pf*ispH* (ID PF3D7_0104400, devoid of BTD; Fig. S1) (amplified with primers 28 and 29), resulting in pRHA1030G-HA-PfIspH. The rhamnose-inducible plasmid was confirmed by Sanger sequencing with primer 30.

For pICOz-based constructs used for transfection of *T. gondii*, plasmid pICOz-UPRT-MCS served as the basis. This construct consists of 750 bp each of *T. gondii'*s 5′- and 3′-UPRT locus (amplified with primers 31/32 and 33/34, respectively, from pGRA-UPRT-TgFd (13)), the endogenous TgFd promoter followed by the BTD of *T. gondii* Fd (synthesized by Biocart), an MfeI site for inserting the different Fd coding sequences, followed by a C-terminal HA tag. All pICOz-based constructs were confirmed by Sanger sequencing with primer 39.

### Disruption of Ec*fldA* by homologous recombination

EcΔ*ispH* strain MgΔLy contains an arabinose-inducible Ec*ispH* copy in addition to the inactivated endogenous *ispH* (*via* insertion of a kanR cassette) on the MG1655 genetic background (30). Knock out of Ec*fldA* was performed according to (32). MgΔLy was first transformed with pMBI followed by the recombineering plasmid pKD78. The donor DNA for knock out was obtained by PCR amplification from an existing *E. coli* strain with the *fldA* gene disrupted by a trimethoprim resistance gene (*tmpR*) (KM20; (19)) and introduced by electroporation. Correct colonies were identified by PCR (with primers 44–47) and verified by proliferation assay (growth in arabinose or Mev-containing media). The strain was finally cured from helper plasmid pKD78. The resulting strain was named EcMP2.

### Complementation of *E. coli* Δ*ispH* strain and bacterial growth assays

*E. coli* MgΔLy containing Pf/Tg*ispH* plasmids were selected on kanamycin (50 μg/ml) and ampicillin (100 μg/ml) plates. A single colony was then used to set up a 5 ml overnight culture at 37 °C. This was used for growth assays in a 96-well plate under the different test conditions ([i] 0.2% arabinose, [ii] 0.2% glucose + 200 ng/ml doxycycline, [iii] 0.2% glucose) along with the antibiotics and allowed to run for 16 h in a prewarmed microplate reader (Tecan Infinite M200 Pro) shaken at an amplitude of 4 mm at 37 °C.

Pf*fnr* was integrated into the genome of EcMP2 using CRIM expression plasmid pLZ42-6x-His-PfFNR at the att80n site as described (89). Correct integration was verified by PCR using primers 40 to 43 and the strain was called EcMP2-F. It was followed by transformation with plasmids expressing PfIspH and PfFd (wt and mutants) (strain EcMP2-FHF). Colonies were selected on agar plates containing tetracycline (15 μg/ml, for pMBI), kanamycin (50 μg/ml), trimethoprim (50 μg/ml), gentamicin (25 μg/ml), chloramphenicol (25 μg/ml), ampicillin (100 μg/ml), and Mev (1 mM). A single colony was used to inoculate 5 ml LB medium containing all the required antibiotics and 1 mM Mev. The overnight preculture was used to set up a secondary culture in medium containing only Mev, ampicillin, and gentamicin, selecting only for the plasmids expressing PfFd and PfIspH. At an A_600nm_ of 0.5 to 0.6, expression was induced with 1 mM IPTG, 200 ng/ml doxycycline and 0.2% rhamnose for 2 h. Thereafter, the A_600nm_ was taken and the cells were washed 3× in PBS. Cell concentrations were adjusted to 2 × 10^4^ cells/μl from which 20 μl was used for each test well. The covered plate was transferred to the pre-warmed microplate reader and incubated as above for up to 50 h.

### Transfection of *T. gondii* tachyzoites

Transfection of TgiΔFd tachyzoites was performed as reported previously using standard techniques (13, 90). To introduce a single copy at a predetermined gene locus in *T. gondii*, CRISPR-Cas9 was used to insert PfFd (wt and mutants) constructs into the UPRT locus using plasmid pSAG1::CAS9-U6::sgUPRT (91). Successful knock-in was selected for by resistance to 5-fluoro-2′-deoxyuridine (FUDR) after electroporation with 50 μg of DNA (40 μg of PfFd plasmid + 10 μg of Cas9 plasmid) in CytoMix using an Amaxa Nucleofector II Device (program T-016; Lonza). Parasites were allowed to lyse before selection with 10 μM FUDR, (Alfa Aesar GmbH). FUDR-resistant clones were then obtained by limiting dilution. gDNA from the clones was PCR amplified with primers 37 and 38. The presence of mutations was verified by Sanger sequencing.

### Plaque assay

The plaque assays (measuring parasite growth over several lytic cycles in the presence of aTc (0.6 μg/ml in ethanol) or vehicle only was performed as detailed previously (92). About 50 parasites/well were used to infect 6-well plates with human foreskin fibroblasts cells and grown for 8 days in Dulbecco's modified Eagle's medium supplemented with 5% fetal bovine serum. Cell monolayers were stained with crystal violet (0.2% dye (Fluka) in 2% ethanol) for 30 to 60 min. Plates were photographed and plaque areas measured using ImageJ 1.53c (https://imagej.net/ij/).

### Immunofluorescence microscopy

*T. gondii* TgiΔFd complemented with PfFd (wt and mutants) were maintained in human foreskin fibroblasts and immunofluorescence assays were performed as described previously (13). The following antibodies were used: Alexa Fluor 546–coupled secondary goat anti-rat antibody (Invitrogen, diluted 1:4000), 4′,6-diamidino-2-phenylindole (Sigma-Aldrich, 1 ng/μl), and Alexa 488–coupled streptavidin (Invitrogen, 1:5000) for localization of biotin containing proteins in the apicoplast (43). Slides were imaged using a Zeiss Axio Imager Z1/Apotome microscope. Images were acquired with a Zeiss AxioCam MRm camera using AxioVision software (Zeiss) and processed using equal linear adjustments for all samples. Image analysis for colocalizations was done using ImageJ 1.53c.

### Western blot analysis

Bacterial cells were lysed in RIPA buffer (150 mM NaCl, 50 mM Tris–HCl, pH 8.0, 1% IGEPAL CA-630, 0.5% sodium deoxycholate, 0.1% SDS, and protease inhibitor cocktail) and sonicated. Protein samples were separated on 12% gels by SDS-PAGE. They were transferred to nitrocellulose membranes by semidry transfer. Total protein on the membrane was stained with removable DB71 dye as described (93) and then blocked for 1 h with 5% skimmed milk in PBS (blocking solution). Immunostaining was performed with mouse anti-His mAb (MAK1396, Linaris; 1:2000), rat anti-HA mAb (3F10, Roche, 1:100), or mouse anti-β-galactosidase (Z378A, Promega, 1: 500) in blocking solution overnight. The membrane was incubated either with anti-mouse horseradish peroxidase (HRP)-linked secondary antibody (Dianova GmbH) at 1:5000 dilution or HRP-conjugated donkey anti-rat secondary antibody (Invitrogen, 1:2000) for 1 h. HRP-derived signal was detected using ECL Plus Western blotting detection reagents (Thermo Fisher Scientific) on a Vilber fusion FX imaging system.

### Metabolomic analysis

EcMP2-FHF transformed with PfFd_wt_ or mutants was first grown for 48 h in 1 mM Mev, ampicillin, and gentamicin to determine the exponential growth phase for sample collection (94). The cells were grown to exponential phase (in 1 mM Mev only, 16 h, A_600nm_ of 0.5–0.7) and proteins induced as described before in the absence of Mev for 2 h. Samples were then taken and snap-frozen in liquid nitrogen after centrifugation at 13000*g* for 1 min to obtain cell pellets. To ensure an equal number of cells (9 × 10^7^ cells), OD_600nm_ was used for protein normalization (1 A_600nm_ = 2.5 × 10^7^ cells/ml).

To establish the metabolite extraction protocol, we adapted methods described in (62), with modifications for our specific experimental conditions. For the metabolite extraction, 50 μl 80% acetonitrile in water was added to the pellet, followed by sonication for 2 min in a cooled isopropanol sonicator bath (Branson Digital Sonifier 450D), and centrifugation at 21500*g*, 0 °C. Supernatants were transferred to vials, 5 μl each were mixed for a pooled biological quality control. A 5 μl aliquot of each sample was injected in a randomized order onto a SeQuant ZIC-pHILIC (Merck) column with an OPTI-LYNX ZIC-pHILIC guard column cartridge (Optimize Technologies) operating on a Vanquish Flex (Thermo Fisher Scientific). Pooled biological quality controls and blanks were injected intermittently. Analytes were chromatographically separated in an isocratic run at 10 mM ammonium carbonate, 118.4 mM ammonium hydroxide, and 60% acetonitrile in water as a mobile phase. Metabolites were detected on a Q Exactive Plus (Thermo Fisher Scientific) equipped with an electrospray ionization source operated in negative mode at an MS 1 resolution of 140 k. Peak extraction was performed with the QualBrowser 3.1 (Thermo Fisher Scientific) or mzMine 2.14 (https://github.com/mzmine/mzmine). Peak annotation was validated by comparison of the *m/z* and retention time using authentic in-house standards. Blank subtraction, gap-filling (at least 50% rule) as well as statistical analysis was performed in Excel (Microsoft). Principal component analysis and hierarchical cluster analysis was performed with ClustVis (95) and GraphPad Prism (version 10.4.2).

### Bioinformatics and software

Protein sequences were retrieved from UniProt (https://www.uniprot.org) and VeuPathDB (https://veupathdb.org), respectively. Phylogenetic analyses were conducted on the NGPhylogeny server (https://ngphylogeny.fr), using modules MAFFT, trimAI, and PhyML+SMS for maximum likelihood-based inference of phylogenies. The resulting tree was constructed with TreeViewer 2.2 (96). Visualization of alignments was performed with JalView 2 (97). 3D protein model predictions were performed online with either AF2 using ColabFold (98) (https://github.com/sokrypton/ColabFold), AF3 (https://golgi.sandbox.google.com/) (35), or the AF3-derivative Chai-1 (https://lab.chaidiscovery.com) which allows the incorporation of ligands into the models (36). For model visualization, ChimeraX 1.9 was used (99). Structural protein alignments were done either within ChimeraX using the MatchMaker module or the Pairwise Structure Alignment tool at the RCSB protein Data Bank (https://www.rcsb.org/alignment) (100). Contacts between protein interfaces of a modeled complex were determined within ChimeraX with the “AlphaFold Contacts” command and/or the Pick Cluster plugin (101), using the respective predicted aligned error for residue pairs. Figures were finalized with Affinity Designer 1.9.

## Statistics

Data analysis and presentation was performed as indicated in the figure legends, with GraphPad Prism (version 10.4.2) and R package ggpubr (https://rpkgs.datanovia.com/ggpubr/).

## Data availability

All data are contained within the article and the Supporting information. Biological material generated in this study is available upon reasonable request by contacting the corresponding author.

## Supporting information

This article contains supporting information. SI references (102, 103, 104, 105, 106, 107, 108, 109, 110, 111, 112, 113, 114, 115, 116, 117, 118, 119, 120, 121, 122, 123, 124, 125, 126, 127, 128).

## Conflicts of interest

The authors declare that they have no conflicts of interest with the contents of this article.
