# Supplementary material for: Reconstituted ferredoxin–MEP pathway of Apicomplexa in Escherichiacoli as an in situ screening platform for inhibitors and essential enzyme mutations
Source: J Biol Chem. 2025 Sep 16;301(11):110726. doi: 10.1016/j.jbc.2025.110726 (PMC12605070; doi:10.1016/j.jbc.2025.110726)
Supplement: Supporting Information [file mmc1.pdf]

Supporting Information

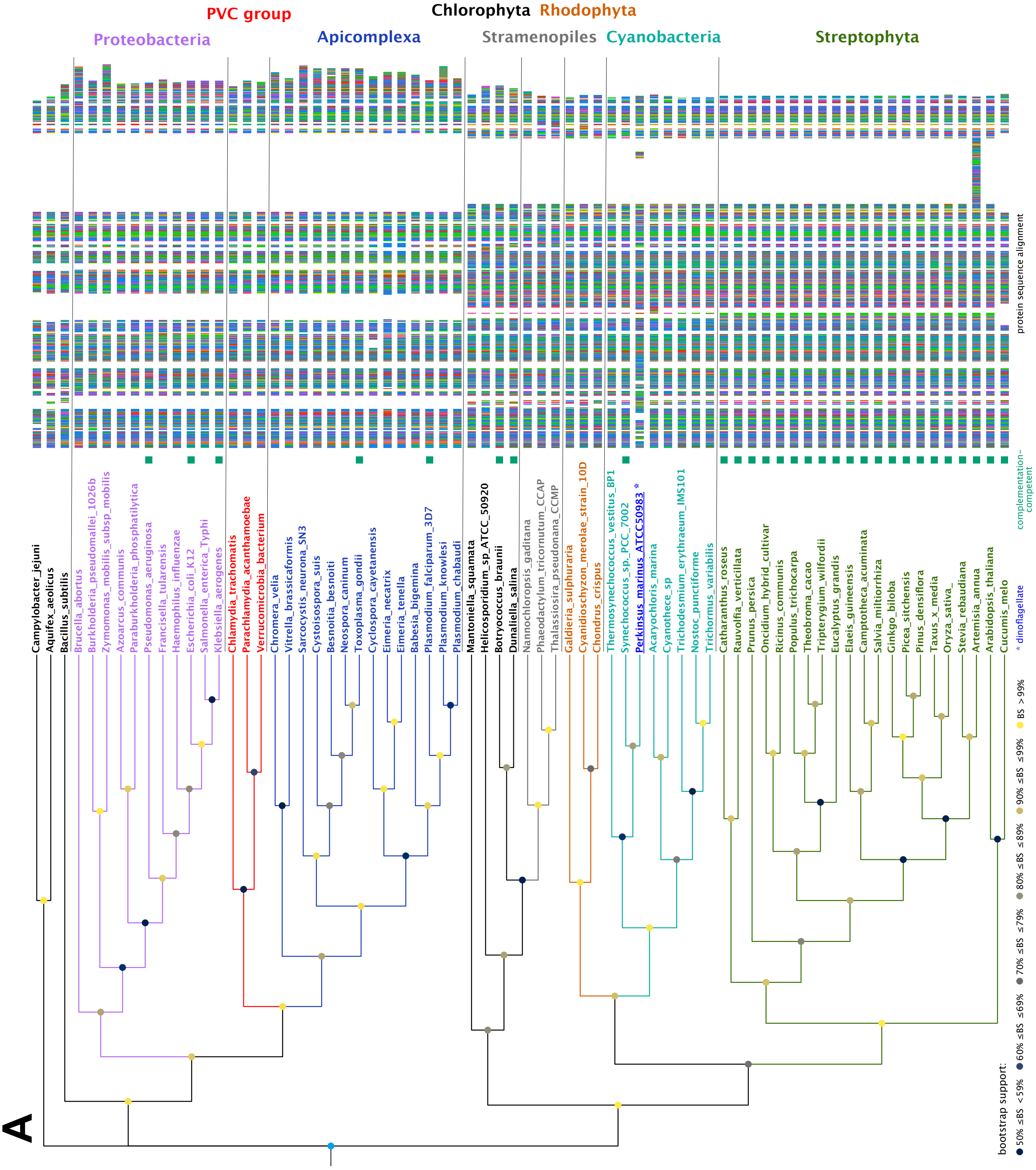

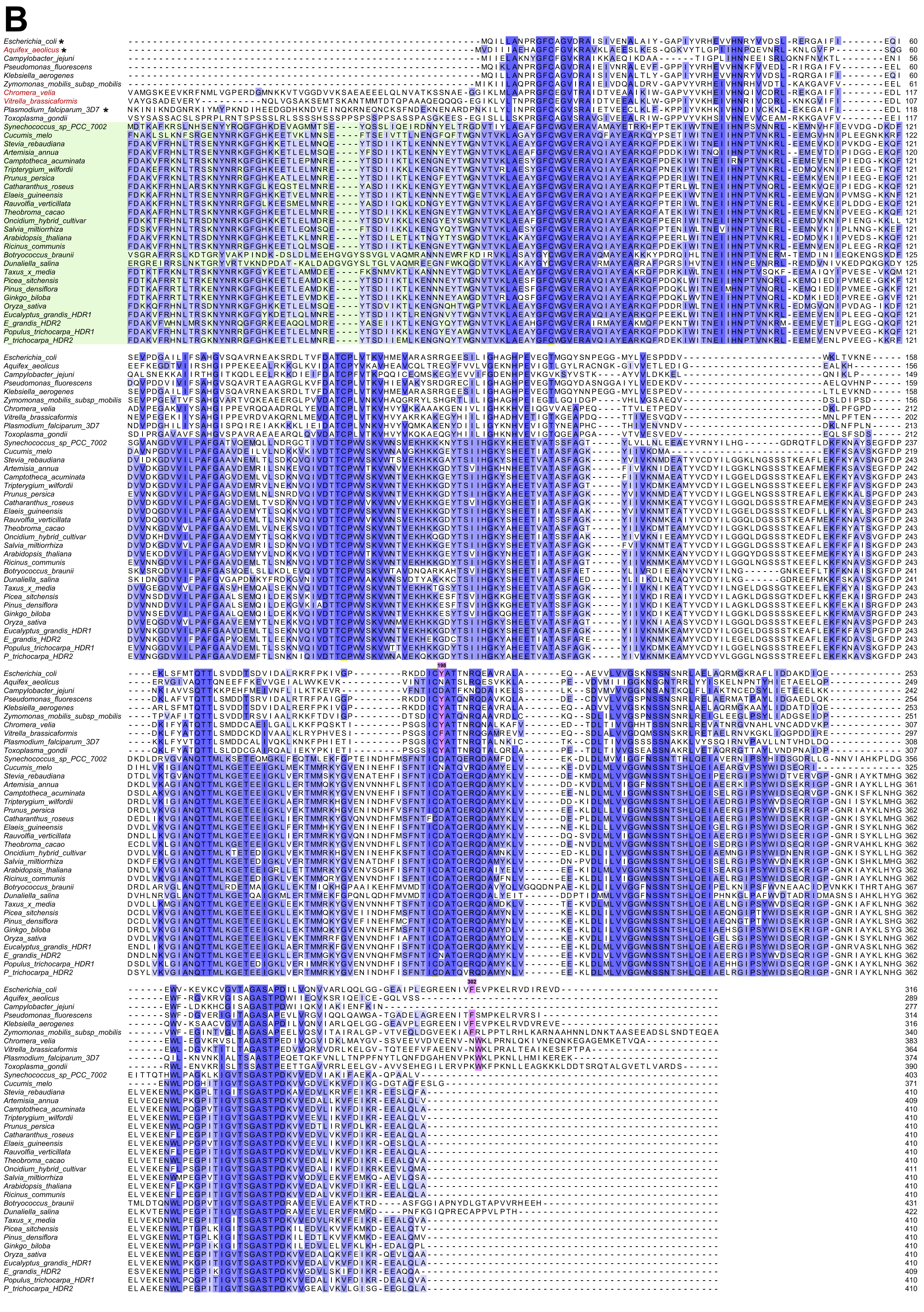

Figure S1. Multiple sequence alignment and phylogenetic analysis of select IspH proteins. *A*, Phylogenetic tree of select IspH proteins. Organisms with complementation-competent IspH sequences are indicated with green rectangles. *B*, MAFFT sequence alignment of all IspH

proteins that have been reported to date to complement an *E. coli* strain with non-functional IspH. Exceptions (red names) are *Aquifex aeolicus, Chromera velia* and *Vitrella brassicaformis*, which are included for comparison. Sequences start with the NCD (green background), as defined by *Synechococcus* IspH. Residues discussed in the text are numbered and highlighted. The cysteines involved in ISC binding are indicated by yellow boxes. Stars mark IspH sequences with solved 3D structures. See table S1 for references.

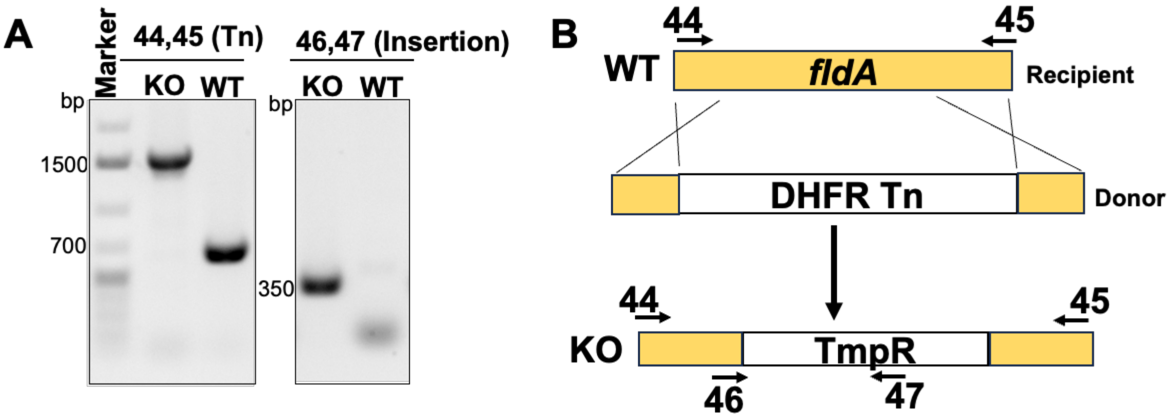

C

Primer pair	WT	KO
44,45 (Tn)	700bp	1500bp
46,47 (Insertion)	-	350bp

Figure S2. Confirmation of Ec*fldA* knockout. *A*, Agarose gel showing molecular confirmation of the KO by PCR. *B*, Outline for the knockout of Ec*fldA* by homologous recombination. *C,* The table shows expected amplicon sizes for the KO and WT with the indicated primer pairs. Insertion of the transposon sequence into *fldA* gene locus causes a disruption of the reading frame, indicative by an increase in amplicon size from around 700 bp in WT to 1.5 kb in the KO (primers 44 & 45). Also, a primer pair designed to show an amplicon only when the transposon was inserted into *fldA* showed an amplicon at the expected size in the KO strain but not in the WT (primers 46 & 47).

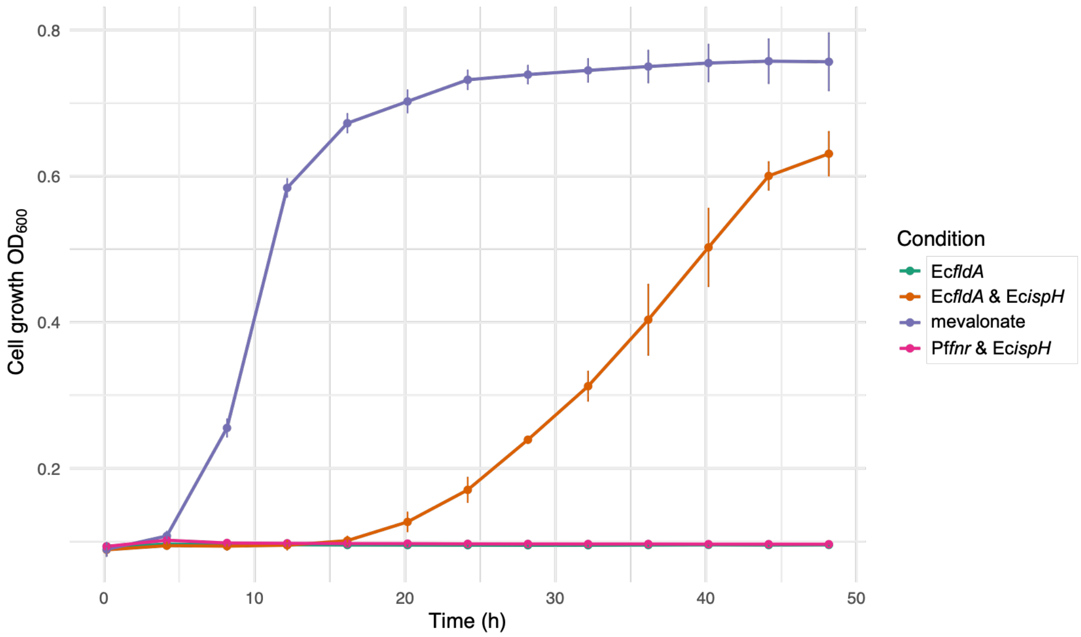

Figure S3. PfFNR cannot reduce EcFldA. The growth assay was performed on EcMP2 with Pf*fnr* integrated into the genome and Ec*fldA* expressed episomally. The culture conditions include rhamnose (to induce Ec*fldA*), rhamnose & arabinose (to induce Ec*fldA* & Ec*ispH* respectively), mevalonate (to induce the by-pass pathway), IPTG & arabinose (to induce Pf*fnr* & Ec*ispH* respectively). Data show a single experiment representative of two independent experiments, each conducted in triplicate. Error bars represent SD and are not visible if smaller than the symbols.

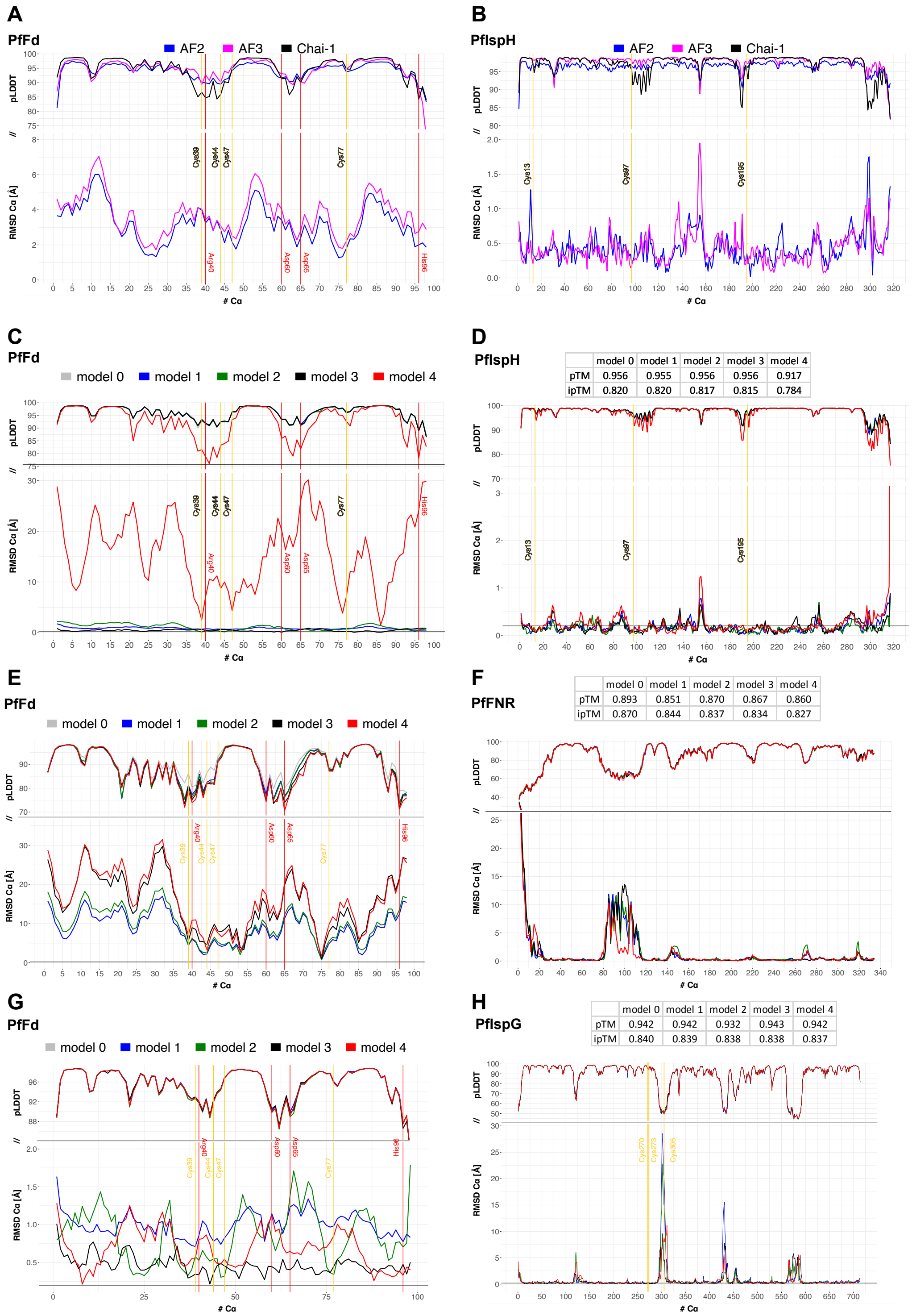

Figure S4. Comparison of models for proteins interacting with PfFd and their general implications. *A,B,* PfFd-PfIspH models were predicted using either AF2 (98) , AF3 or Chai-1, and root mean square deviation (RMSD) and predicted local distance difference test (pLDDT) values of the highest-ranked model of each prediction were compared along the amino acid (# Cα) to allow the visualization of deviations from each 3D structure (RMSD) in 2D. For this, the root mean square deviation (RMSD) per residue as well as the predicted Local Distance Difference Test (pLDDT) values were extracted from MatchMaker-generated .pdb files using custom scripts. The differences of RMSD compared to the highest ranked model as reference were plotted together with the corresponding pLDDT along the residue number as a line chart in R (ggpubr package). Higher RMSD at a position means more deviation of the respective Cα atom to the reference model. The positions of the Cys residues involved in ISC interaction as well as

five essential mutant amino acid are indicated. Note that our modelling was only intended to illustrate that the experimental data are in accordance with those predictions, i.e. we did not for example, perform extensive repetitions of runs (102). In essence, all three algorithms resulted in similar models, but since Chai-1 allows the incorporation of any ligands, it is easier to validate the models via the positions of ISCs and the substrate and thus distances required for optimal electron flow. *C-H*, Similar analysis as in *A,B* for PfFd-PfIspH (*C,D*), PfFd-PfFNR (*E,F)* and PfFd-PfIspG (*G,H)*. Here, the five returned models of a prediction with Chai-1 are compared to model 0 (highest rank) as reference. Overall, it can be seen that RMSD variation in PfFd is low in the PfFd-PfIspG complex and higher in the other two but that the Cys residues and thus the ISC position is less affected.

In the context of the PfFd-PfFNR model, it is interesting to note that in a previous genome-wide association study of *P. falciparum* field isolates, a $PfFd_{D97Y}$ mutation had been suspected to be associated with resistance to the antimalarial drug artemisinin (103). Subsequent *in vitro* studies with this mutant and its interaction with PfFNR concluded that the mutant "could be associated with the action of artemisinin" (80). However, when others introduced the $PfFd_{D97Y}$ mutation into transgenic *P. falciparum* blood stage parasites, it did not translate into altered artemisinin sensitivity (104). Not only does this illustrate the limitations of *in vitro* experiments, it also cautions against overinterpreting static AF3-based models, particularly when the affected amino acid is located in the more flexible terminal part of a protein (105,106).

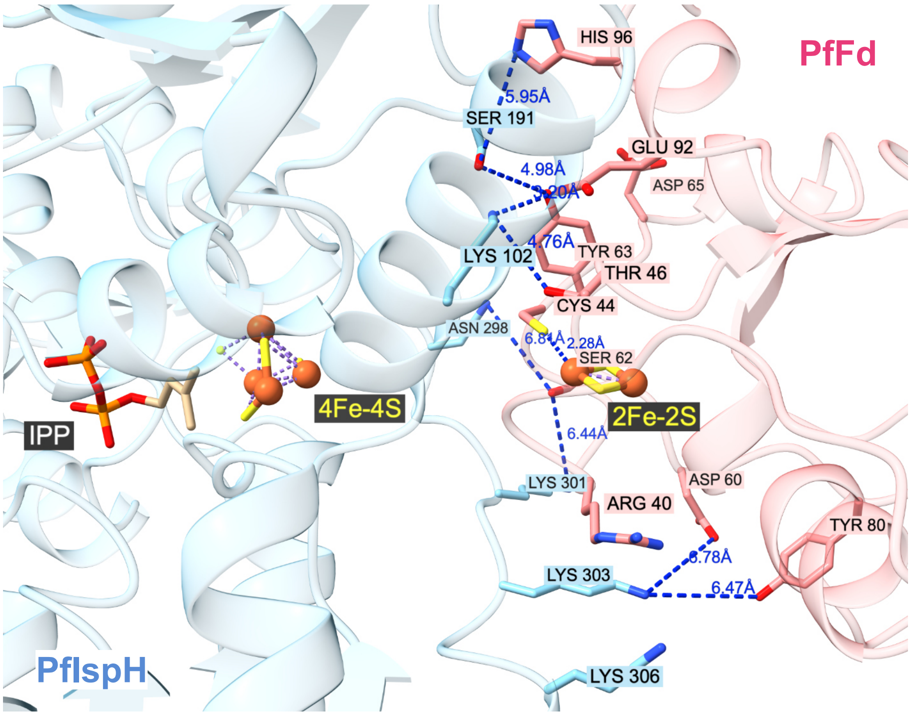

Figure S5. Predicted interaction interface between PfFd and PfIspH.
A, Predicted interaction interface between PfFd (light red) and PfIspH (light blue), based on Chai-1 model. The selected residues of PfFd for mutagenesis as well as the likely interacting amino acids on PfIspH are shown, together with some calculated distances. The 4-ISC cluster and the ligand HMBPP of PfIspH and the 2-ISC cluster of PfFd are also drawn as ball-and-stick structures.

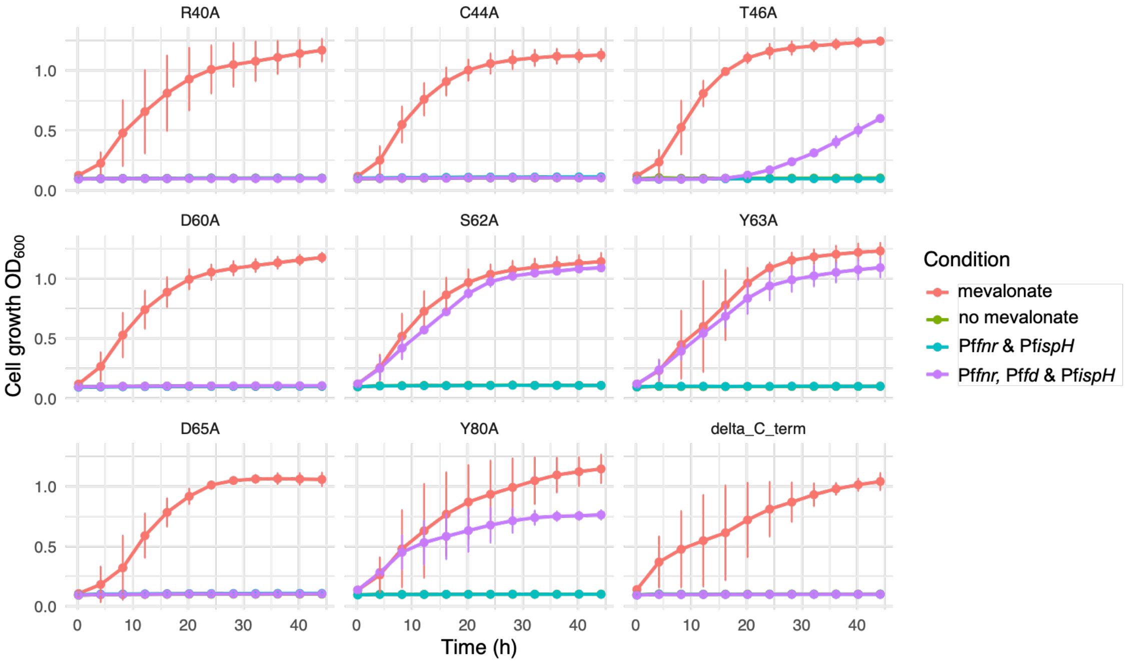

Figure S6. Growth curve for each of the PfFd mutants in Fig. 6. The culture conditions include IPTG & rhamnose (to induce Pf*fnr* & Pf*ispH* respectively), IPTG, doxycycline & rhamnose (to induce Pf*fnr,* Pf*fd* & Pf*ispH* respectively), mevalonate (to induce the by-pass pathway), or no mevalonate (no-induction control). Values are averaged from three independent experiments (n = 3), error bars represent SD and are not visible if smaller than the symbols.

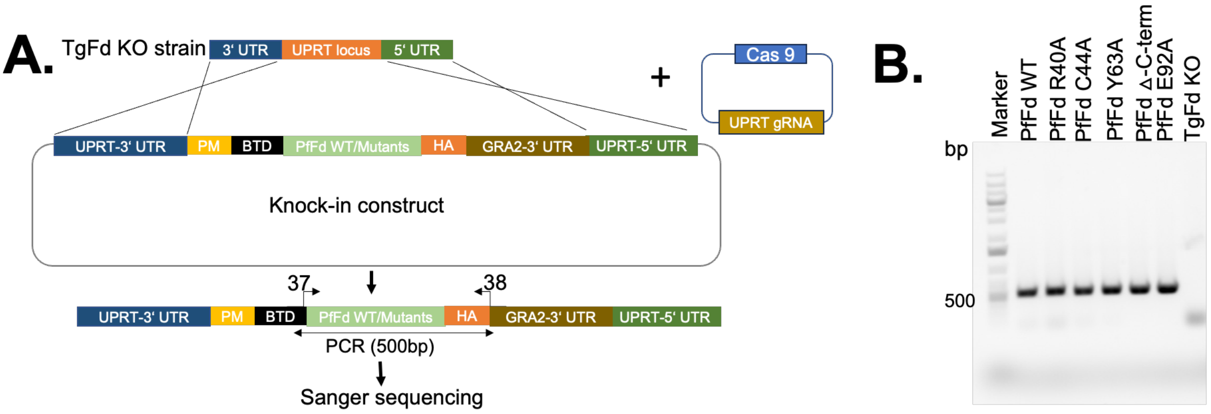

Figure S7. Knock-in of $PfFd_{WT}$ and mutants into the UPRT locus of TgiΔFd. *A,* Schematic representation of the knock-in of $PfFd_{WT}$ or mutants into the endogenous UPRT locus using a Cas9 expressing plasmid with UPRT-specific gRNA. Loss of UPRT activity upon integration of the construct is selected for by FUDR selection (13,107). The knock-in construct has an HA tag at the C-terminus of the PfFd insert in frame with GRA2-3'UTR for mRNA stability, proper processing and efficient translation of the insert. PM= TgFd promoter; BTD= Bipartite targeting domain of TgFd. 37 & 38 represent the primers used for PCR and Sanger sequencing and can be found in table S2. *B,* Agarose gel of PCR showing successful knock-in of the inserts. Correctness (presence of mutation) of the amplicons were further verified by Sanger sequencing.

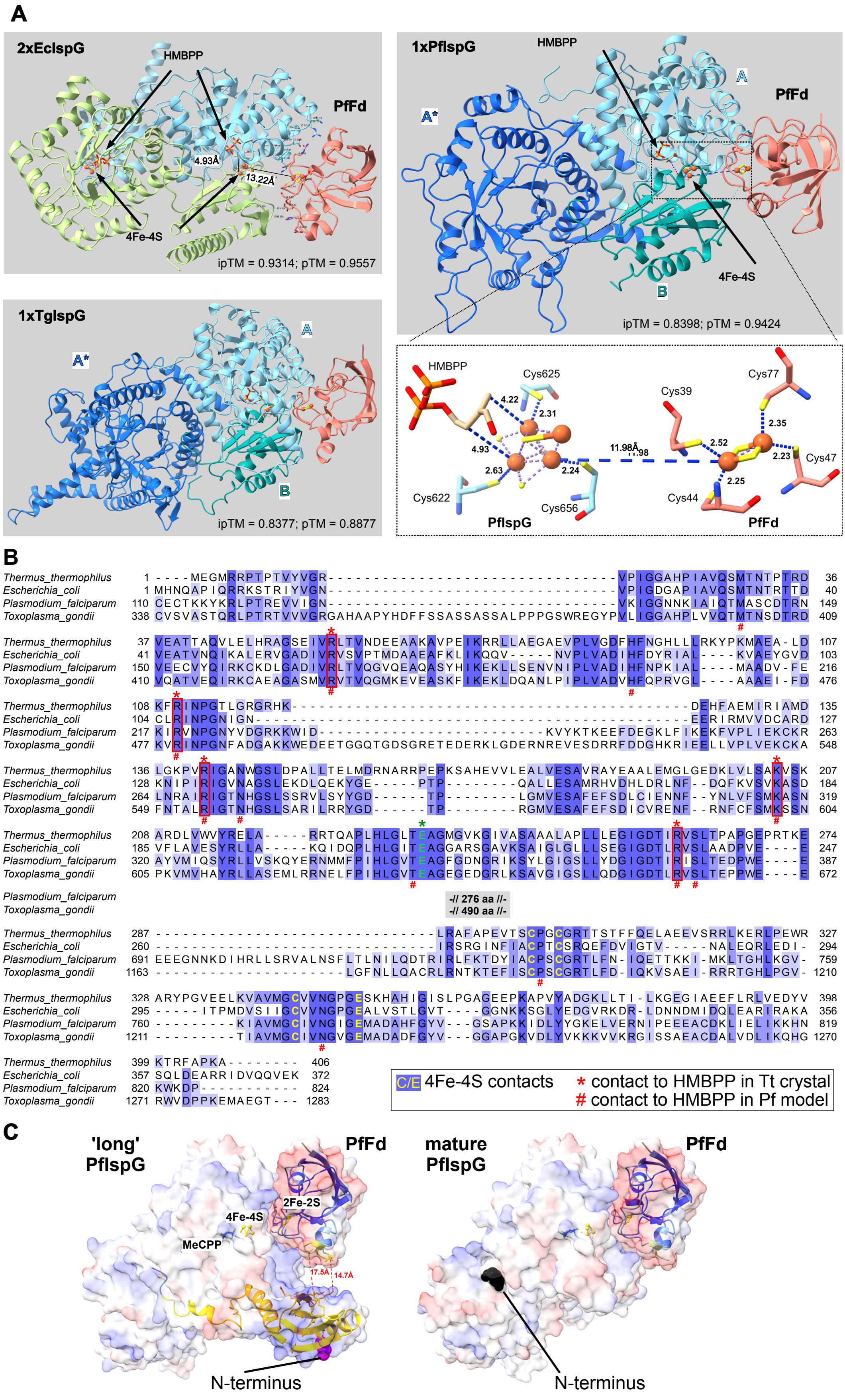

Figure S8. IspG models and protein sequences. *A,* Chai-1 model of head-to-tail homodimeric *E. coli* IspG in complex with PfFd (top left). Only a head-to-tail homodimer is able to bring the 4-ISC cluster in

close enough proximity (≤14 Å) to the ligand (HMBPP) to allow efficient electron transfer (108) (*top left*), consistent with the experimentally determined *Thermus thermophilus* 3D structure (109,110). PfIspG is more than 300 amino acids longer (Fig. S7B), which allows its folding into a monomeric structure containing a third domain (termed A*) (45) and that it is presumably active without dimerization (*top and bottom right*), as had been suggested previously for plant and *P. falciparum* IspG (44). The inferred relative positions of the interacting ligands and the lengths between atoms which correspond to literature values required for proper functioning support this model: PfFd's 2Fe-2S ⇔ PfIspG's 4Fe-4S: < 4Å (111); PfIspG's 4Fe-4S ⇔ PfIspG's HMBPP < 14Å (108). *B*, Multiple sequence alignment of IspG proteins, with the two apicomplexan proteins being shortened at their N-termini to match the bacterial IspG start. Grey box indicates the number of inserted amino acids in PfIspG and TgIspG. Functionally important amino acids known from the *T. thermophilus* 3D structure are indicated. *C*, Chai-1 model of PfIspG, from its start methionine to the end ('long' PfIspG; *left*) in comparison to the model starting with aa 110 (see *B*) (mature PfIspG; *right*). These extra 109 aa contain 24 positively charged residues. In both models, the electrostatic potential (blue, positive; red, negative) of the surfaces is shown. Two exemplary distances between aa of long PfIspG (K42, K59) and PfFd (D65, D97) are drawn. In addition, the cartoon structure of the 109 aa (colored by pLDDT score) of long PfIspG is given. Ligands as well as the N-terminal aa of the respective model are indicated.

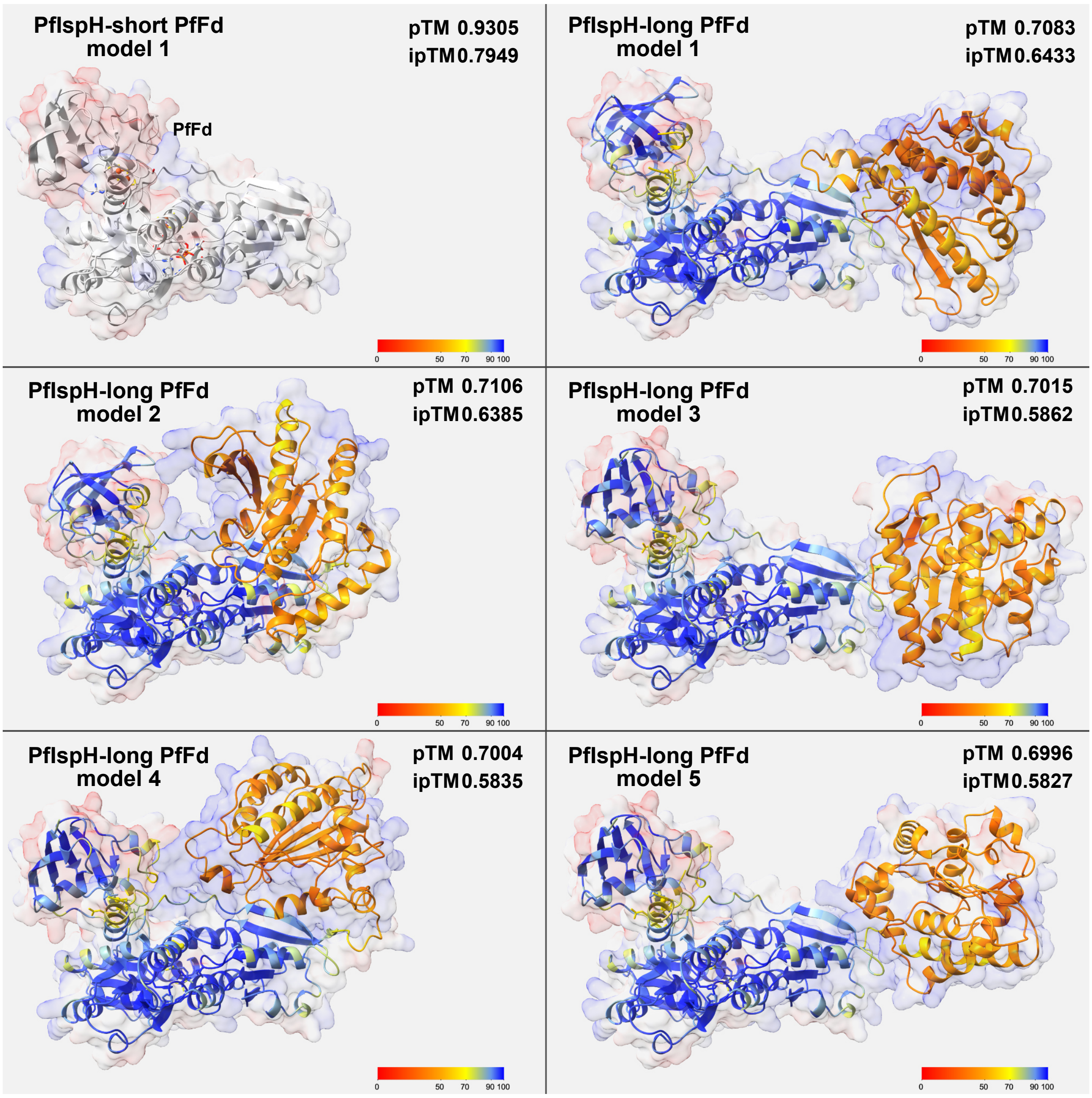

Figure S9. Comparison of models of PfIspH with its extra N-terminal aa in complex with PfFd. The top left model is based on the sequence defined in Fig. S1 and in the text. The ChimeraX color key reflects the pLDDT values from 100 (very high confidence) to 0 (no confidence) per residue. The electrostatic potential (blue, positive; red, negative) of the respective surfaces is indicated. Five Chai-1 complex predictions (with all ligands included) for the long protein are shown. They were aligned using MatchMaker.

Given the lack of either experimental data or reliable prediction algorithms able to suggest cleavage sites of the BTD, the entire coding sequence was used for the long version. Compared to the short version, the longer N-terminus results in much lower pTM and ipTM values, and pLDDT scores are overall below 50 (orange to red coloring), indicating low confidence in the model's predictions of the N-termini. In model 2 and 4, the overall positively charged N-terminus is in relative proximity to PfFd. Whether this has an influence on the interaction with PfFd is unknown.

Table S1: Studies on the complementation of *E. coli* Δ*ispH* and Δ*ispG* with *ispH/ispG* genes from plants and other bacteria

Organism	Reference
Arabidopsis thaliana	(112)
Arabidopsis thaliana	(71)
Artemisia annua	(113)
Botryococcus braunii	(114)
Camptotheca acuminata	(115)
Cucumis melo	(116)
Dunaliella salina	(117)
Ginkgo biloba	(118)
Oncidium sp.	(119)
Oryza sativa	(120)
Pinus densiflora	(121)
Rauvolfia verticillata	(122)
Salvia miltiorrhiza	(123)
Stevia rebaudiana	(124)
Taxus media	(125)
Tripterygium wilfordii	(126)
Ricinus communis	(73)
Populus trichocarpa	(73)
Prunus persica	(73)
Eucalyptus grandis	(73)
Theobroma cacao	(73)
Elaeis guineensis	(73)
Picea sitchensis	(73)
Synechococcus sp. PCC7002	(73)
Arabidopsis thaliana	(73)
Enterobacter aerogenes	(56)
Pseudomonas fluorescens	(56)
Campylobacter jejuni	(127)
Zymomonas mobilis	(128)
Arabidopsis thaliana	(70) *
Catharanthus roseus	(69) *

*IspG complementation

Table S2: Cloning primers

Primer number	Primer sequence (5'-3')
1	GTTTAACTATAAGAAGGAGATATACATATGGCGCACCATCACCATCATCATGCCG GCATTTCGTTGCTTCTTTC
2	GACTCTAGAGGATCCCCGGGTACCATGGTTAAGAGTCTCGGGCGACCAG
3	TAAGCTTGACCTGTGAAGTGAAAAATGGCG
4	AGCGTAGTCCGGAACGTCATACGGGTACATTTGTATATCTCCTTCTTAAAGTTAAA C
5	CTTTAAGAAGGAGATATACAAATGTACCCGTATGACGTTCCGGACTACGCTATGG CTTTTTACAACATTACC
6	CATTTTTCACTTCACAGGTCAAGCTTACATATCGTGTAATTCATCCTC
7	CGAATGGCCAGATGATTAATTC
8	GTACTCATGCGCCGGCGGGTCGTG
9	GGCAGTTCTACGTTCTGAC
10	GTCGTGTTCCGCCTGTGCGGCAA
11	CCGCCCCGGCATGAGTAC
12	CGATGACCAGGCCTATCTCGATG
13	TTGTCAACTTCACCTTCC
14	TGACAACGATGCCCAGTCTTATC
15	ACTTCACCTTCCACCAGT
16	TGACCAGTCTGCCCTCGATGAGG
17	TCGTTGTCAACTTCACCTTC
18	GTCTTATCTCGCCGAGGAACAAATCAAAAAG
19	TGGTCATCGTTGTCAACTTC
20	GTGTACGTGCGCCCCAAAAAGTGATTG
21	AGAAGAATGTATTTCTTTTTGATTTG
22	GGGCGGGTCGGCCTCCACCTGTG
23	CGGCATGAGTACGGCAGT
24	CATCGAAACATAAAAAGAGGATGAATTAC
25	ACGCAATCACTTTTTGGATAG
26	TCATGTTTGACAGCTTATCATCGATGGTCAGTATTGAGCGATATCTAG
27	ACCATGGTGAATTCCTCCTGCTAGCTTATTGCTCAGCGGTGGCG
28	GTTTAACTATAAGAAGGAGATATACATTACCCGTATGACGTTCCGGACTACGCTA TGGCAAATAAGATCCTGTACCTC
29	CTAGAGGATCCCCGGGTACCATGGTCACTCGAGTTTCTCGCGCTC
30	CATGTTTGACAGCTTATC
31	CATTTCTCTCGCCTCCACGTTTTTCTCGAATTCTCCTGGTCGTCTGTCTC
32	ACAGAGACAGACGACCAGGAGAATTCGAGAAAAACGTGGAGGCGAGAG
33	TGCGCATCACTTTCGTCGTAGTCTTAATTAACTACGCGTAGTCCGGGAC
34	GTCCCGGACTACGCGTAGTTAATTAAGACTACGACGAAAGTGATGCGCA
35	CGTCCGTCTCCGAAGTCACTCGCGCCTATTTTACAACATTACCTTGCGC
36	CGCGTAGTCCGGGACGTCGTACGGGTACCAATTCATATCGTGTAATTCATCCTC
37	CTTGTCTCGGAGCTGCGCAGAG
38	CTGATCGGCTTTGTAGACTTCTC
39	GCTGGACGCAGAGACGGTGC
40	CTGCTTGTGGTGGTGAAT
41	ACTTAACGGCTGACATGG
42	ACGAGTATCGAGATGGCA
43	TAAGGCAAGACGATCAGG

44	GATTGCCTTTATCCGTGGGC
45	AGCTGTTCACCTTTGGCACT
46	GTAGCACAGTGTGCAGTCCTG
47	CGTCGAGATGCAACTCTTCAG
